# Circadian rhythm related genes identified through tumorigenesis and immune infiltration-guided strategies as predictors of prognosis, immunotherapy response, and candidate drugs in skin cutaneous malignant melanoma

**DOI:** 10.3389/fimmu.2025.1513750

**Published:** 2025-03-21

**Authors:** Chengling Liu, Xingchen Liu, Pengjuan Cao, Haiming Xin, Xin Li, Sailing Zhu

**Affiliations:** ^1^ Center of Burns and Plastic Surgery and Dermatology, The 924th Hospital of Joint Logistics Support Force of the Chinese People's Liberation Army (PLA), Guilin, China; ^2^ Department of Pathology, The First Affiliated Hospital of Naval Medical University, Shanghai, China; ^3^ Department of Endocrinology and Traditional Chinese Medicine, The 924th Hospital of Joint Logistics Support Force of the Chinese People's Liberation Army (PLA), Guilin, China

**Keywords:** melanoma, circadian rhythm related genes, prognosis, tumor immune microenvironment, immunotherapy

## Abstract

**Background:**

Skin cutaneous malignant melanoma (SKCM) is among the most aggressive forms of skin cancer, notorious for its rapid progression and poor prognosis under late diagnosis. This study investigates the role of circadian rhythm-related genes (CRGs) in SKCM addressing a gap in understanding how CRGs affect tumor progression and patient outcomes.

**Methods:**

An analysis of CRGs expression was conducted on SKCM samples derived from The Cancer Genome Atlas datasets(TCGA). Moreover, a correlation between various subtypes and their clinical features was identified. The study employed various bioinformatics methods, including differential expression analysis, consensus clustering, and survival analysis, to investigate the role of CRGs. The functional consequences of various CRG expression patterns were further investigated using immune infiltration analysis and gene set variation analysis (GSVA). A scoring system based on CRGs was developed to predict overall survival (OS) and treatment responses in SKCM patients. The predictive accuracy of this score system was then tested, and a nomogram was used to improve its clinical usefulness.

**Results:**

Key findings from this study include significant genetic alterations in circadian rhythm-related genes (CRGs) in skin cutaneous melanoma (SKCM), such as mutations and CNVs. Two molecular subtypes with distinct clinical outcomes and immune profiles were identified. A prognostic model based on six CRGs (*CMTM*, *TNPO1*, *CTBS*, *UTRN*, *HK2*, and *LIF*) was developed and validated with TCGA and GEO datasets, showing high predictive accuracy for overall survival (OS). A high CRGs score correlated with poor OS, immune checkpoint expression, and reduced sensitivity to several chemotherapeutics, including AKT inhibitor VIII and Camptothecin.

**Conclusions:**

This work provides valuable insights into the circadian regulation of SKCM and underscores the potential of CRGs as biomarkers for prognosis and targets for therapeutic interventions. The novel molecular subtypes and CRGs prognostic scoring model introduced in this study offer significant contributions to the understanding and management of SKCM.

## 1 Introduction

Cutaneous malignant melanoma (SKCM) is a highly aggressive skin cancer originating from melanocytes. In recent years, the incidence of SKCM has been on the rise, particularly in Western countries, where it has become one of the fastest-growing malignancies. Recent cancer statistics indicate that in 2023, the United States is projected to see approximately 97,610 new melanoma cases and 7,990 associated fatalities (1). Projections suggest a substantial increase, with potential rises to 510,000 new cases and 96,000 deaths by 2040 if current trends persist (2). Although early detection and treatment could significantly improve survival rates, the prognosis remains poor once metastasis occurs, with a low five-year survival rate (3). Immunotherapy has emerged as a key therapeutic option for metastatic melanoma since the development of immune checkpoint inhibitors, such as anti-PD-1 and anti-CTLA-4 antibodies (4). However, despite the significant therapeutic effects observed in some patients, a considerable proportion does not respond to treatment or eventually develop resistance, greatly limiting the widespread clinical application of immunotherapy (5). Therefore, identifying reliable biomarkers to predict patient responses to immunotherapy and to develop personalized treatment strategies is of paramount importance.

Circadian rhythm genes (CRGs) regulate various physiological processes by controlling the cell cycle, DNA repair, and metabolism (6). In recent years, increasing research has revealed the crucial role of CRGs in cancer development, progression, and treatment response (7). Dysregulated CRG expression is strongly linked to tumor growth and a poor prognosis in many forms of cancer. For example, studies have shown that changes in CRG expression in breast cancer could affect cell proliferation and apoptosis, thereby influencing patient outcomes (8). Although research on CRGs in melanoma is relatively limited, existing evidence suggests that these genes might play a role in tumor development by regulating the tumor microenvironment, immune evasion, and metabolic reprogramming (9). Thus, exploring the mechanisms by which CRGs function in melanoma, particularly in the context of immunotherapy, holds significant theoretical and clinical implications.

Recent studies have increasingly illuminated the crucial role of CRGs in the field of cancer immunotherapy, emphasizing their potential to revolutionize treatment modalities for diseases like melanoma, which is heavily dependent on immunological strategies for management and cure (10). Specifically, CRGs might influence tumor responses to immunotherapy by regulating the circadian rhythms of the immune system. For instance, key CRGs such as CLOCK and BMAL1 have been shown to modulate the functional state of immune cells within the tumor microenvironment, thereby affecting the efficacy of immunotherapy (11). This modulation is crucial because it can considerably improve immunotherapy efficacy by matching treatment time to the patient’s biological clocks, possibly enhancing therapeutic effectiveness while reducing side effects. Moreover, the expression patterns of CRGs might be closely related to patient prognosis, offering possibilities for the development of prognostic models based on CRGs. In melanoma, given its strong reliance on immunotherapy, focusing on how CRGs affect melanoma’s response to immunotherapy, this research could pave the way for novel, more effective treatment protocols that are synchronized with the patient’s circadian biology, offering a new dimension to oncological care and a promising avenue for future scientific exploration and clinical application.

Building on this background, this study aims to systematically analyze the expression characteristics of CRGs and their relationships with immunotherapy response and patient prognosis by integrating clinical and gene expression data from a large cohort of SKCM patients. We hypothesize that CRGs expression patterns predict patient responses to immunotherapy, and serve as independent prognostic markers, providing new foundations for the precision treatment of melanoma. The results of this study are expected to support personalized treatment strategies for melanoma and expand the application prospects of CRGs in cancer immunotherapy.

## 2 Materials and methods

### 2.1 Data acquisition and circadian rhythm-related genes

The Cancer Genome Atlas (TCGA) (https://portal.gdc.cancer.gov/), the Genotype-Tissue Expression Project (GTEx) (https://www.genome.gov/Funded-Programs-Projects/Genotype-Tissue-Expression-Project), and the Gene Expression Omnibus (GEO) (https://www.ncbi.nlm.nih.gov/geo) databases provided data on gene expression and clinical pathology for Skin Cutaneous Melanoma (SKCM). All samples in the datasets were derived from Homo sapiens, with patients lacking survival information excluded. To enhance the consistency and reliability of the data, we integrated gene expression data from the TCGA-SKCM (The Cancer Genome Atlas - Skin Cutaneous Melanoma) and GSE65904 datasets. Specifically, the TCGA provided FPKM (Fragments Per Kilobase of transcript per Million mapped reads) sequencing data for 471 SKCM patients, combined with corresponding clinical information, which served as an essential foundation for our subsequent analyses (12) (**Table 1**), and transcriptome data for 812 normal skin samples from the GTEx database (13). The GSE65904 dataset, based on the GPL10558 platform, comprises transcriptome data from 214 SKCM patients of Homo sapiens origin (14, 15). During the processing of dataset GSE65904, normalization was performed by applying a log transformation to the expression data to eliminate skewness and enhance comparability. Additionally, the avereps function was used to average genes with repeated measurements, thereby reducing technical noise. Standardization was carried out using the normalizeBetweenArrays function from the limma package (16) to adjust for technical variations between samples, ensuring data consistency and comparability. These steps ensured the reliability and accuracy of the subsequent analysis results. During the data integration process, differences in experimental conditions, technical platforms, and sample handling methods between datasets may lead to expression biases, known as batch effects. These inconsistencies could negatively affect the subsequent analysis results. Therefore, we applied the “Combat” algorithm to correct for batch effects in the data. The Combat algorithm uses parametric modeling to evaluate and adjust the impact of batch effects on gene expression data, thereby eliminating systematic biases between batches. This correction ensured that data from different sources could be compared under the same standard, providing a more robust foundation for downstream analyses. After correcting for batch effects, we generated a merged transcriptomic dataset that effectively eliminated potential inconsistencies caused by data discrepancies, ensuring the accuracy and reliability of statistical analyses. This process is crucial for subsequent analyses, including differential expression analysis, gene correlation studies, and prognostic evaluations. The TCGA-SKCM dataset’s FPKM values were translated to transcripts per million (TPM). After merging the TCGA-SKCM and GSE65904 datasets, the “Combat” method was used to eliminate batch effects (**Supplementary Figure S1**).

**Table 1:** Baseline data from TCGA.

Characteristic	levels	TCGA-SKCM
n		471
Gender, n (%)	Female	179 (38%)
	Male	292 (62%)
Age, n (%)	<=60	252 (54.4%)
	>60	211 (45.6%)
T stage, n (%)	T1	41 (11.3%)
	T2	79 (21.7%)
	T3	91 (25%)
	T4	153 (42%)
N stage, n (%)	N0	235 (56.8%)
	N1	74 (17.9%)
	N2	49 (11.8%)
	N3	56 (13.5%)
M stage, n (%)	M0	418 (94.4%)
	M1	25 (5.6%)
Pathologic stage, n (%)	Stage I	77 (18.7%)
	Stage II	140 (34%)
	Stage III	171 (41.5%)
	Stage IV	24 (5.8%)
OS event, n (%)	Alive	247 (53.2%)
	Dead	217 (46.8%)
DSS event, n (%)	Alive	267 (58.3%)
	Dead	191 (41.7%)
PFI event, n (%)	Alive	153 (33%)
	Dead	311 (67%)

To investigate somatic mutations, 469 SKCM patients’ “Masked Somatic Mutation” data were chosen from the TCGA GDC website. VarScan software was used to preprocess the data, and the maftools program was used to show somatic mutations (17). We downloaded the “Masked Copy Number Segment” data for 471 SKCM patients in order to examine copy number variations (CNVs) in important genes among TCGA-SKCM patients. GISTIC 2.0 was used to evaluate the CNV segment data (18). In GenePattern (https://cloud.genepattern.org) to investigate CNVs in circadian rhythm-related genes (CRGs).

Circadian rhythm-related key genes (CRGs) were obtained from previous studies and reviews (19, 20). Aiming to represent the core regulatory roles of circadian rhythms in various biological processes, we extracted 17 CRGs for further research, including *ARNTL*, *ARNTL2*, *CLOCK*, *CRY1*, *CRY2*, *CSNK1D*, *CSNK1E*, *NPAS2*, *NR1D1*, *NR1D2*, *PER1*, *PER2*, *PER3*, *RORA*, *RORB*, *RORC*, and *TIMELESS*. In this study, the selection of 17 CRGs was based on their well-established roles in circadian regulation and cancer research. Circadian rhythm genes are highly conserved throughout evolution, from invertebrates to humans, and play critical roles in regulating physiological functions and disease-related pathways. The same foundational principle applies to melanoma, as circadian rhythm disruptions can influence cancer cell cycles and growth (19). Therefore, the selection of these genes primarily considered their functional conservation in model organisms. Additionally, the impact of these genes in prostate cancer, particularly their key roles in the tumor immune microenvironment, was also taken into account (20). Our selection process incorporated similar bioinformatics analyses, including the use of TCGA and GeneCards databases, along with Lasso and Cox regression analyses, to confirm the expression patterns of these genes in melanoma samples and their alignment with circadian rhythm regulation characteristics. Special attention was given to genes that have demonstrated significant influence on tumor progression and immune regulation in multiple studies. As a result, the selected CRGs not only represent key molecules in the circadian regulation of melanoma but may also influence melanoma growth and development by modulating the tumor immune microenvironment. The selection of these genes was guided by their demonstrated significant biological functions in prior research, with the aim of further uncovering the roles of circadian rhythms in tumor progression.

### 2.2 Differential expression, gene correlation, and prognosis analysis

To further investigate the expression characteristics of circadian rhythm-related genes (CRGs) in SKCM samples, we integrated the TCGA-SKCM and GTEx datasets and performed differential expression analysis between the tumor group (SKCM) and the normal group (normal skin tissues) using the limma package. During the data analysis, we evaluated the expression levels of each gene and identified those significantly upregulated or downregulated in tumor tissues. The results of the differential expression analysis were presented as boxplots, which visually depict the significant differences in gene expression between the two groups. Subsequently, to explore the relationships among CRGs, we performed Spearman rank correlation analysis. This method calculates correlation coefficients between gene expression levels, revealing significant correlations and regulatory networks among the genes. Using the ggplot2 package for visualization, we generated a correlation heatmap that clearly illustrates the expression patterns among different CRGs, providing valuable insights into their potential biological functions. Based on the expression levels of CRGs in SKCM, we further divided the samples into a low-expression group (0%-50%) and a high-expression group (51%-100%). This grouping strategy facilitates the analysis of how different expression levels of CRGs impact patient prognosis. Subsequently, we used the survminer and survival packages to analyze the prognostic differences between these two groups. We applied a Cox regression model to evaluate survival risks between the low-expression and high-expression groups and used the log-rank test to compare the survival curves between groups.

### 2.3 Identification of circadian rhythm-related molecular subtypes

One technique for figuring out the size and composition of possible clusters in a dataset (gene expression profiles) is consensus clustering (21). In order to distinguish between distinct circadian rhythm-associated subtypes, we used the “ConsensusClusterPlus” package to conduct consensus clustering on the combined TCGA and GEO datasets utilizing important genes linked to circadian rhythm (21). ClusterAlg = “pam” and distance = “euclidean” were used to sample 80% of the total data in 100 repetitions, with the number of clusters being set between 2 and 9.

### 2.4 Relationship between circadian rhythm-related molecular subtypes and clinical features

We examined the connections between molecular subtypes, clinical pathological features, gene expression levels, molecular functions, and immunological infiltration in order to investigate the clinical use of the two subtypes found by consensus clustering. Patient characteristics included age, gender, and TNM staging. Additionally, we downloaded the “c5.go.bp.v7.5.1.symbols” and “c2.cp.kegg.v7.4.symbols” gene sets from the MSigDB database (22) to perform gene set variation analysis (GSVA). Gene expression matrices from various samples were converted into gene set expression matrices using GSVA, a non-parametric and unsupervised analytic technique, in order to assess gene set enrichment findings from transcriptome microarrays (23). Based on the gene expression matrix of each sample, pathway scores were computed using the GSVA package in R (https://github.com/rcastelo/GSVA), and the limma program was used to perform differential screening of enriched functions or pathways.

Additionally, we measured the relative abundance of immune cell infiltration for every sample using the ssGSEA method (24). There are 28 different kinds of immune cells that have been discovered, including natural killer T cells, macrophages, activated CD8 T cells, and activated B cells. The relative abundance of each kind of immune cell in the samples was represented by enrichment scores that were computed using ssGSEA. The ggplot2 software was used to visualize the association between immune cell expression and several molecular subtypes.

### 2.5 Identification of differentially expressed genes (DEGs) between molecular subtypes

Using the R limma package, differential analysis of genes between several molecular subtypes was carried out. The criterion of a fold change more than 1.5 and an adjusted p-value less than 0.05 was used to identify differentially expressed genes (DEGs) among subtypes of circadian rhythms. In order to maximize the inclusion of DEGs with practical biological significance in terms of expression levels, we selected |logFC| > 0.585 and adjusted P value < 0.05 as the screening criteria (25). DEGs that were found to be up-regulated were those with logFC > 0.585 and adj p value < 0.05, whereas down-regulated DEGs were identified as those with logFC < -0.585 and adj p value < 0.05.

### 2.6 Functional enrichment analysis of differentially expressed genes between circadian rhythm subtypes

A popular technique for conducting extensive functional enrichment studies on biological processes (BP), molecular functions (MF), and cellular components (CC) is gene ontology (GO) analysis (26). The Kyoto Encyclopedia of Genes and Genomes (KEGG) is an extensive database that stores data about illnesses, medications, biological processes, and genomes (27). Using the clusterProfiler package (28), GO annotation analysis and KEGG pathway enrichment analysis of DEGs were carried out (27), with FDR < 0.05 being regarded as statistically significant. Adj p.value < 0.05 and q.value < 0.05 were the selection criterion for entries, and the Benjamini-Hochberg technique (BH) was used to correct the p-value.

### 2.7 Identification of circadian rhythm-related gene clusters and construction of a prognostic model

To further search prognostic genes and construct a prognosis model, multivariate Cox regression analysis, lasso regression analysis, and univariate Cox regression analysis were used. Initially, univariate Cox regression analysis was carried out on DEGs, keeping genes linked with SKCM prognosis if p < 0.05. Consensus clustering was used to classify SKCM patients into distinct gene clusters, and then these gene clusters were examined for variations in gene expression and prognosis. Based on the combined TCGA and GEO datasets, all SKCM patients were randomly divided into training and test sets in a 1:1 ratio. The LASSO technique (29) was utilized in the training set to reduce multicollinearity and filter significant variables from univariate Cox regression analysis. To acquire more accurate independent prognostic indicators (prognostic characteristic genes), we applied multivariate Cox regression analysis with stepwise regression for final screening. Lastly, by considering the optimized gene expression and corresponding estimated Cox regression coefficients, we generated a risk score formula: Riskscore=(exp−Gene1×coef−Gene1)+(exp−Gene2×coef−Gene2)+…+(exp−Gene×coef−Gene) . The training and test set samples were separated into high-risk and low-risk categories based on their median risk score (30). The term exp-Gene represents the expression level of a specific gene, and coef-Gene represents the regression coefficient of that gene. To compare overall survival between the training and test sets, Kaplan-Meier analysis was performed using the survival program. Furthermore, survival prediction was assessed using time-dependent receiver operating characteristic (ROC) curves, and prognostic or predictive accuracy was measured by calculating the area under the ROC curve (AUC) using the timeROC R package (31).

### 2.8 Gene Set Enrichment Analysis

We employed Gene Set Enrichment Analysis (GSEA) to perform functional enrichment analysis in order to investigate biological process differences between high-risk and low-risk SKCM patients. A computer technique called GSEA assesses if a predetermined gene set exhibits statistically significant changes between two biological states (32). Usually, it is applied to quantify changes in biological process and pathway activity within samples of expression datasets. For Gene Set Enrichment Analysis, the gene sets “c2.cp.v7.2.symbols.gmt” and “h.all.v7.2.symbols.gmt” were obtained from the MSigDB database (22), with a false discovery rate (FDR) < 0.25 regarded as substantially enriched.

### 2.9 Immune infiltration analysis

CIBERSORTx (33) is a method that estimates the makeup and quantity of immune cells inside mixed cells by deconvolution of transcriptome expression matrices using linear support vector regression. To generate the immune cell infiltration matrix, we uploaded gene expression matrix data (TPM) to CIBERSORTx, used the LM22 signature gene matrix, and filtered samples with p<0.05. The association between risk scores, critical genes, and immune cell infiltration levels was investigated. The ESTIMATE R package (34) was utilized to predict stromal and immune cell scores from gene expression profiles, followed by the calculation of these cell counts. We investigated the relationship between ESTIMATE scores and high- and low-risk categories.

### 2.10 Prediction of immunotherapy response and drug sensitivity

Utilizing the Tumor Immune Dysfunction and Exclusion (TIDE) tool (35), prognosis was assessed based on risk scores, and variations in TIDE, Dysfunction, and Exclusion scores between high-risk and low-risk groups were evaluated. Additionally, immunotherapy responses in SKCM patients were predicted. Genomic sensitivity indicators and tumor drug response information were found using the Genomics of Drug Sensitivity in Cancer (GDSC) database (36). Using the pRRophetic algorithm (37), we constructed a ridge regression model based on the expression profiles of CCLE cell lines and TCGA-SKCM gene expression profiles. This allowed us to estimate the IC50 values for the sensitivity of common anticancer treatments in both high-risk and low-risk groups.

### 2.11 Construction of a nomogram scoring system

We conducted univariate and multivariate Cox regression studies on risk scores and clinical parameters in order to optimize the prediction power of the model and further evaluate the influence of risk scores and clinicopathological aspects on patient prognosis. The R package “regplot” (https://CRAN.R-project.org/package=regplot) was used to construct a nomogram by integrating clinical features and risk scores, predicting 1-, 3-, and 5-year survival probabilities for SKCM patients. Each variable in the nomogram scoring system has a corresponding score, and the sum of all the variable scores for each sample yields the final score. After that, time-dependent ROC curves and calibration curves were used to assess the nomogram’s discriminating power.

### 2.12 Statistical analysis

**Figure 1:**
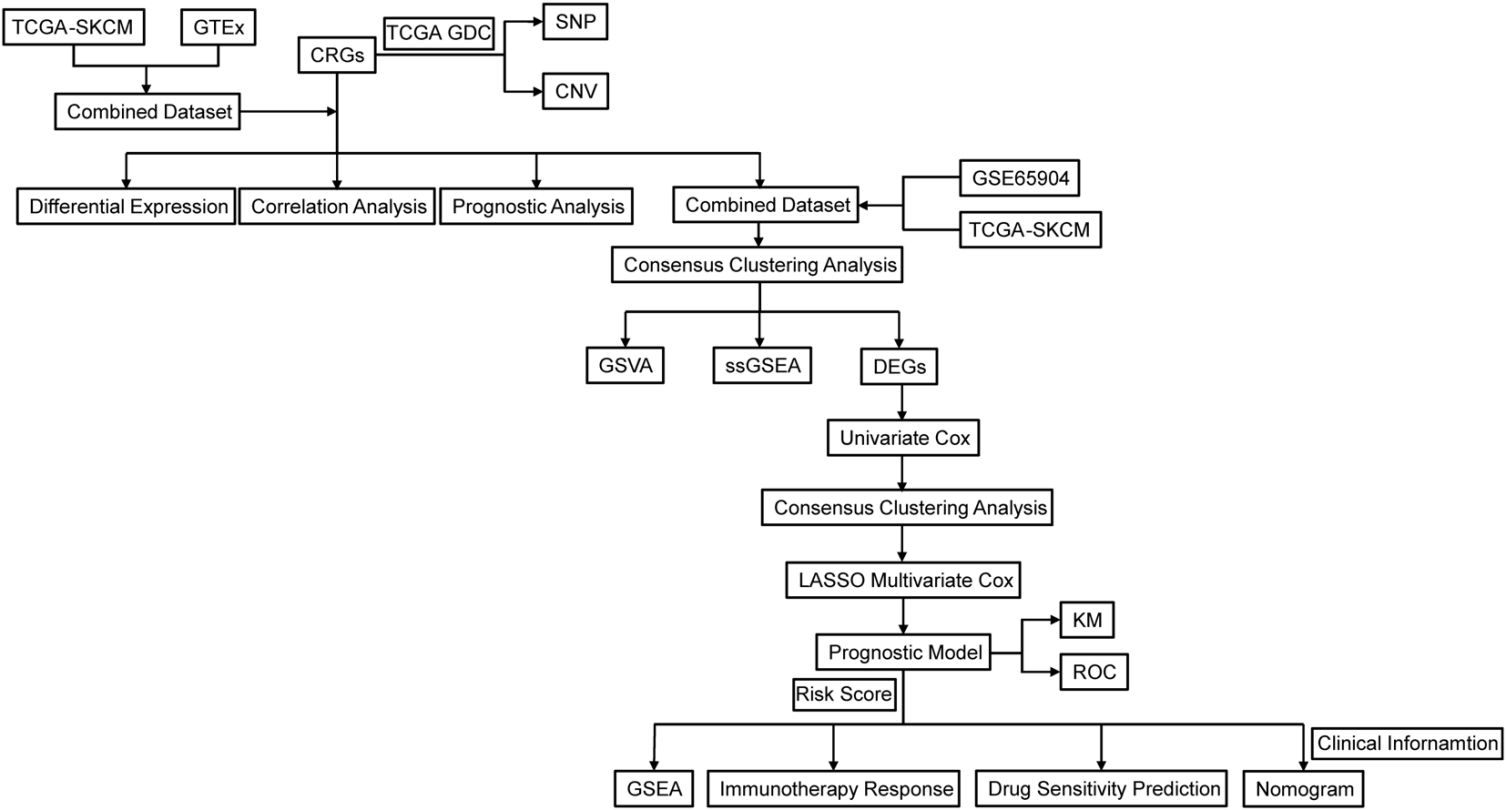
Flowchart of the research. TCGA, The Cancer Genome Atlas; GDC, Genomic Data Commons; SKCM, Skin cutaneous malignant melanoma; CRGs, circadian rhythm-related genes; GTEx, Genotype-Tissue Expression Project; SNP, Single Nucleotide Polymorphism; CNV, copy number variations; GSVA, gene set variation analysis; ssGSEA, single sample gene set enrichment analysis; DEGs, Differentially Expressed Genes; LASSO, Least Absolute Shrinkage and Selection Operator; KM, Kaplan-Meier; ROC, Receiver Operating Characteristic Curve.

R programming was used for all statistical analysis and data processing. The Mann-Whitney U test (also known as the Wilcoxon rank-sum test) was used to analyze differences between non-normally distributed variables, while the independent Student’s t-test was used to estimate the statistical significance of normally distributed variables for comparisons between two groups of continuous variables. P < 0.05 was deemed statistically significant for all two-sided statistical p-values. A detailed flow chart of this research can be found in **Figure 1**.

## 3 Results

### 3.1 SNPs and CNVs in circadian rhythm-related genes

**Figure 2:**
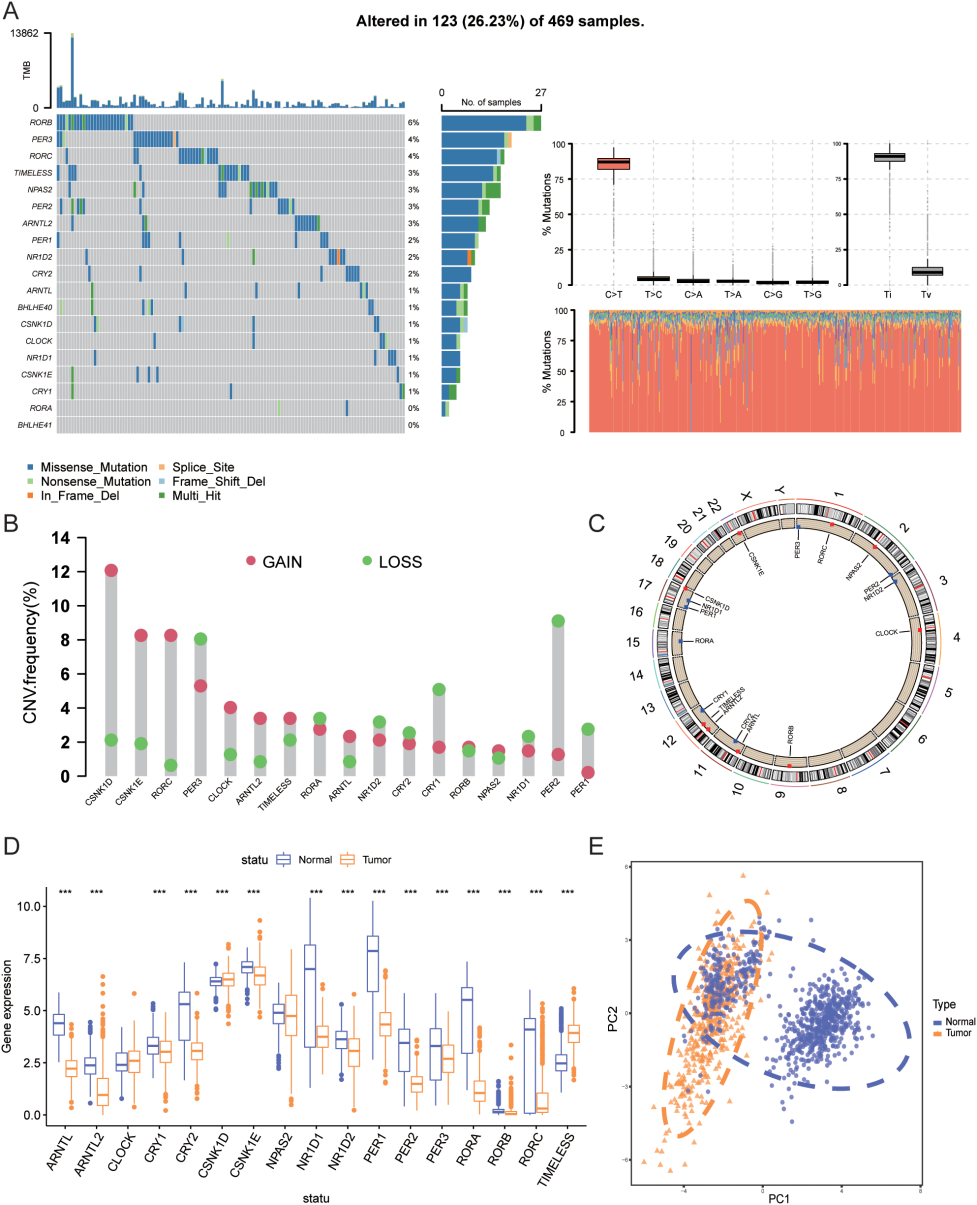
Genetic and transcriptomic alterations of CRGs. **(A)** Mutation frequencies of 17 CRGs in patients from TCGA. **(B)** Frequency of CNV gains and losses among CRGs. **(C)** Locations of CNV changes of CRGs across 23 chromosomes. **(D)** Expression differences of 17 CRGs between normal tissues and SKCM tissues. **(E)** PCA results of transcriptomic data from tumor and normal tissues. *** indicates P<0.001. TCGA, The Cancer Genome Atlas; CRGs, Circadian Rhythm Related Genes; CNV, Copy Number Variations; SKCM, Skin Cutaneous Melanoma; PCA, Principal Component Analysis.

This study included 17 circadian rhythm-related genes (CRGs). A summary analysis of somatic mutation rates in these 17 CRGs revealed that 123 out of 469 SKCM samples (26.23%) had mutations in CRGs. Among these, *RORB* had the highest mutation frequency (6%), followed by *PER3*, *RORC*, *TIMELESS*, and others (**Figure 2A**). Next, we investigated somatic copy number variations (CNVs) in these CRGs (**Figure 2B**) and found that CNVs were common across all 17 CRGs. Notably, *CSNK1D*, *CSNK1E*, *RORC*, and *CLOCK* exhibited widespread increases in copy number variations, whereas *PER3*, *PER2*, *PER1*, and *CRY1* showed decreases in CNVs. **Figure 2C** illustrates the locations of CNV changes in CRGs on their respective chromosomes, such as CNV changes in *CSNK1D* located on chromosome 17 and in *CSNK1E* on chromosome 22.

### 3.2 Differential expression, gene correlation, and prognosis analysis

We further compared the mRNA expression levels of CRGs between tumor tissues and normal tissues. As shown in **Figure 2D**, compared with normal skin tissues, the expression of *ARNTL*, *ARNTL2*, *CRY1*, *CRY2*, *CSNK1E*, *NR1D1*, *NR1D2*, *PER1*, *PER2*, *PER3*, *RORA*, *RORB*, and *RORC* was significantly downregulated in tumor tissues (P<0.001), whereas the expression of *CSNK1D* and *TIMELESS* was significantly upregulated (P<0.001). **Figure 2E** shows the PCA analysis results of tumor and normal tissues.

**Figure 3:**
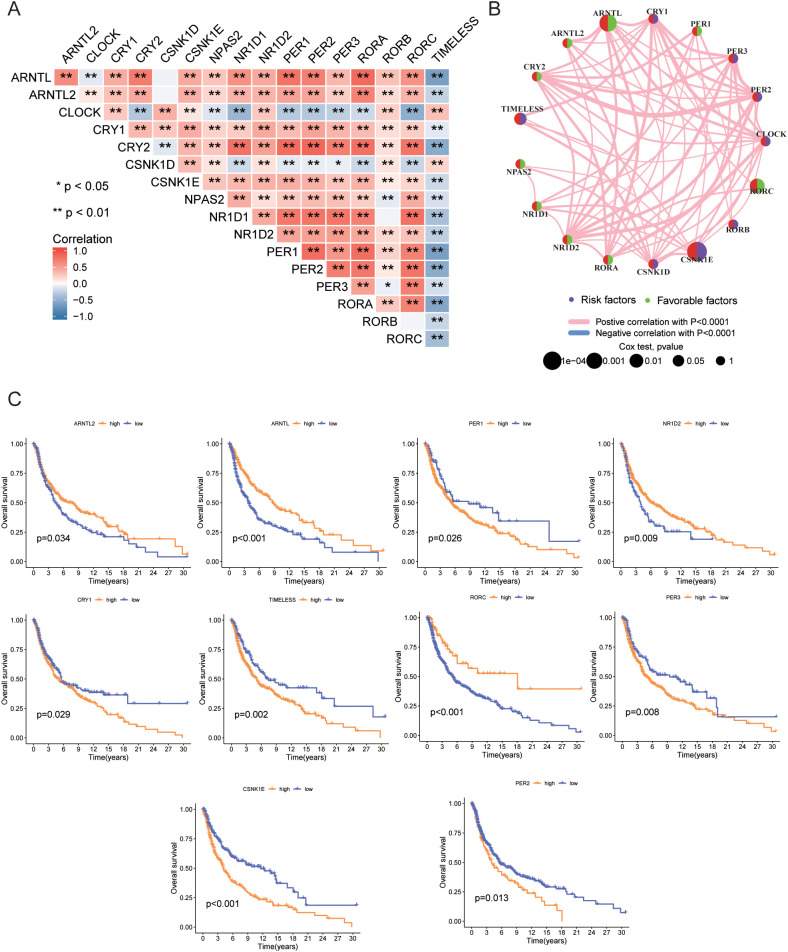
Gene correlation and prognosis analysis. **(A)** Correlation of expression levels among 17 CRGs. **(B)** Prognostic network diagram. **(C)** Prognostic differences between high and low expression groups of CRGs based on TCGA data. • indicates P<0.05, ** indicates P<0.01, *** indicates P<0.001. CRGs, Circadian Rhythm Related Genes; TCGA, The Cancer Genome Atlas.

Next, we analyzed the expression correlation among CRGs. Spearman correlation analysis revealed significant correlations between the expression of the 17 genes, with most genes showing positive correlations, except for *TIMELESS* and *CLOCK*, which were negatively correlated with other genes (**Figure 3A**). **Figure 3B** illustrates the interactions among 17 CRGs, with each node representing a gene and edges denoting the correlations between genes and their associations with patient prognosis. The color and style of the edges indicate the type of correlation: pink lines represent positive correlations, while blue lines represent negative correlations, both with high statistical significance (P < 0.0001). The network reveals that most CRGs exhibit significant positive correlations, suggesting co-expression in tumor cells and involvement in related biological processes. In contrast, negative correlations involving TIMELESS, CLOCK, and other genes indicate distinct functions or regulatory mechanisms, potentially playing opposing roles in tumor progression. This network provides insight into the complex interplay of CRGs and their roles in melanoma biology. Moreover, **Figure 3C** highlights the association between gene expression and patient prognosis, demonstrating that high expression of ARNTL2, ARNTL, NR1D2, and RORC correlates with favorable outcomes, while elevated levels of PER1, PER2, CRY1, TIMELESS, PER3, and CSNK1E are linked to poor prognosis. By illustrating both the interactions among these genes and their impact on survival time and quality of life, the figure provides valuable insights into the prognostic significance of specific gene expressions and the complex interactions of CRGs and their roles in melanoma biology. Understanding the interactions between these genes and their prognostic significance may provide new biomarkers for clinical applications, facilitating risk stratification and personalized treatment strategies.

### 3.3 Identification of circadian rhythm-related molecular subtypes

**Figure 4:**
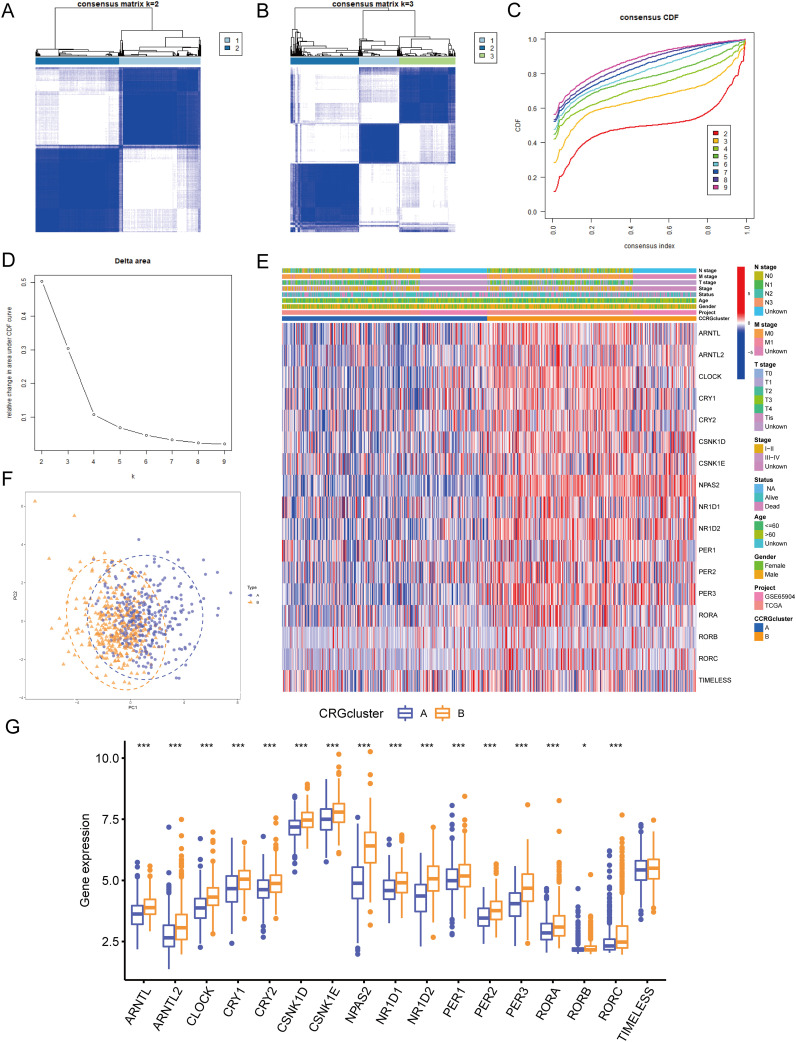
Identification of circadian rhythm-related molecular subtypes. **(A)** Consensus clustering heatmap defining two clusters (k = 2) and their associated area. **(B)** Consensus clustering heatmap defining three clusters (k = 3) and their associated area. **(C)** CDF plot showing the distribution of consensus clustering corresponding to each k **(D)** Delta area plot visualizing the relative change in the area under the CDF curve at different k values. **(E)** Heatmap showing the relationship between gene expression levels and clinicopathological parameters across different clusters. **(F)** PCA analysis showing differences in gene expression profiles between the two subtypes. **(G)** Expression differences of CRGs between CRG cluster A and B. • indicates P<0.05, ** indicates P<0.01, *** indicates P<0.001. CDF, Cumulative Distribution Function; PCA, Principal Component Analysis; CRGs, Circadian Rhythm Related Genes.

In this study, to identify circadian rhythm-related molecular subtypes of melanoma, we employed an unsupervised consensus clustering analysis. Specifically, we used the “ConsensusClusterPlus” R package to perform consensus clustering on the combined TCGA and GEO datasets, with 17 circadian rhythm-related genes as features. This method assesses the stability of different clustering numbers by repeated resampling and uses the consensus matrix to evaluate the optimal number of clusters (k). Ultimately, we determined that k=2 was the optimal clustering number and categorized the patients into two molecular subtypes accordingly. **Figure 4A** shows the consensus matrix generated through consensus clustering analysis, where the color intensity of each cell represents the similarity between samples. For the clustering scenario with k=2, the samples are divided into two main groups, illustrating the relationships between the samples and the concentration of their clustering. **Figure 4B** The figure displays the consensus matrix for k=3. Compared to k=2, the sample division becomes more complex, potentially resulting in multiple subgroups. At this point, the similarity between samples decreases, indicating a lower clustering quality and suggesting that the clustering may not be optimal under these conditions. **Figure 4C** presents the consensus CDF curves for different k values. Compared to other k values, the curve for k=2 has the largest proportion in the region above 0.8, indicating it as the optimal clustering choice. This provides strong evidence for determining the most suitable number of clusters for the samples. **Figure 4D** shows the number of clusters (k) on the x-axis and the change in clustering quality (Delta area) on the y-axis. As the k value increases, there is a clear downward trend in the Delta area, with the most significant drop observed at k=2. This further supports the conclusion that k=2 is the optimal choice. **Figures 4A–D** display the clustering results obtained at different k values based on the consensus clustering method. After considering the consensus cumulative distribution function (CDF) curve, Delta area variation, and clustering stability, we ultimately selected k=2 as the optimal number of clusters. This classification was based on the similarity of gene expression patterns between samples, rather than traditional methods using mean or median cutoffs, ensuring the rationality and stability of the SKCM molecular subtypes. Significant variations in the gene expression patterns of the two subtypes were found using PCA analysis (**Figure 4F**). **Figures 4E, G** show the relationship between CRG expression and clinicopathological features, with circadian rhythm-related genes expressed significantly higher in CRG cluster B than in CRG cluster A: *ARNTL* (P<0.001), *ARNTL2* (P<0.001), *CLOCK* (P<0.001), *CRY1* (P<0.001), *CRY2* (P<0.001), *CSNK1D* (P<0.001), *CSNK1E* (P<0.001), *NPAS2* (P<0.001), *NR1D1* (P<0.001), NR1D2 (P<0.001), *PER1* (P<0.001), *PER2* (P<0.001), *PER3* (P<0.001), *RORA* (P<0.001), *RORB* (P<0.05), *RORC* (P<0.001).

### 3.4 GSVA and immune infiltration analysis

**Figure 5:**
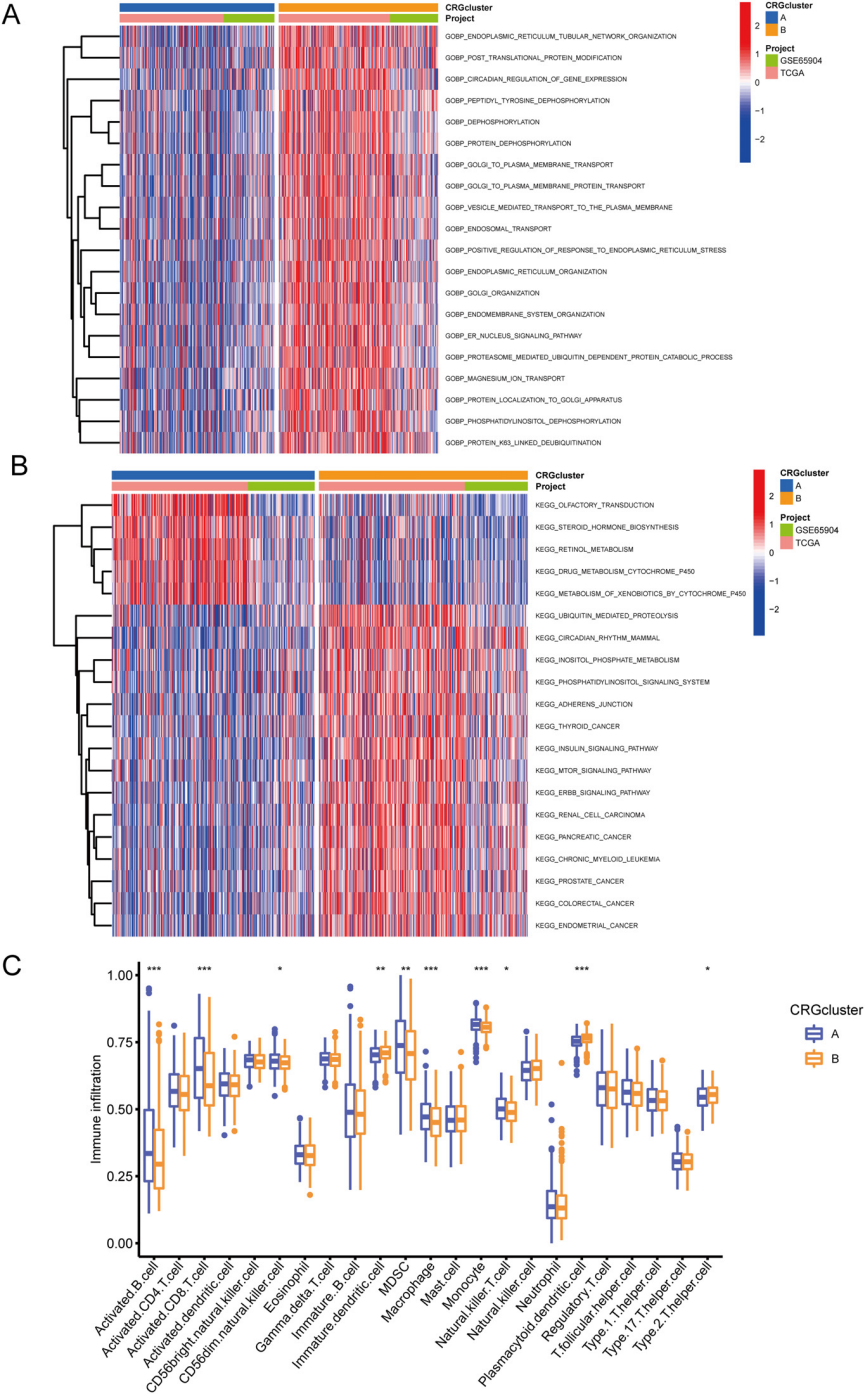
GSVA and immune infiltration analysis. **(A)** GSVA-GOBP analysis results between CRG cluster A and B. **(B)** GSVA-KEGG analysis results between CRG cluster A and B. **(C)** ssGSEA immune infiltration analysis showing differences in the relative abundance of immune cells between CRG cluster A and B. • indicates P<0.05, ** indicates P<0.01, *** indicates P<0.001. GSVA, Gene Set Variation Analysis; GOBP, Gene Ontology Biological Process; KEGG, Kyoto Encyclopedia of Genes and Genomes.

GSVA enrichment analysis indicated that CRG cluster B was significantly enriched in biological processes related to protein synthesis, metabolism, and substance transport, including positive regulation of response to endoplasmic reticulum stress, golgi organization, magnesium ion transport, and er nucleus signaling pathway (**Figure 5A**). Additionally, cluster B was enriched in pathways such as mtor signaling pathway, erbb signaling pathway, insulin signaling pathway, and retinol metabolism (**Figure 5B**). To further investigate the role of CRGs in the tumor immune microenvironment (TME), we used the ssGSEA algorithm to assess the relative abundance of immune cells in each SKCM sample and the differences between clusters (**Figure 5C**). The results showed that the immune infiltration levels of activated B cells, activated CD8+ T cell, CD56dim natural killer cells, Myeloid-derived suppressor cells, macrophages, monocytes, and natural killer T cells were significantly higher in CRG cluster A than in CRG cluster B. In contrast, immature dendritic cells, plasmacytoid dendritic cells, and type 2 T helper cells were significantly more infiltrated in CRG cluster B than in CRG cluster A.

### 3.5 Identification of differentially expressed genes (DEGs) between molecular subtypes

**Figure 6:**
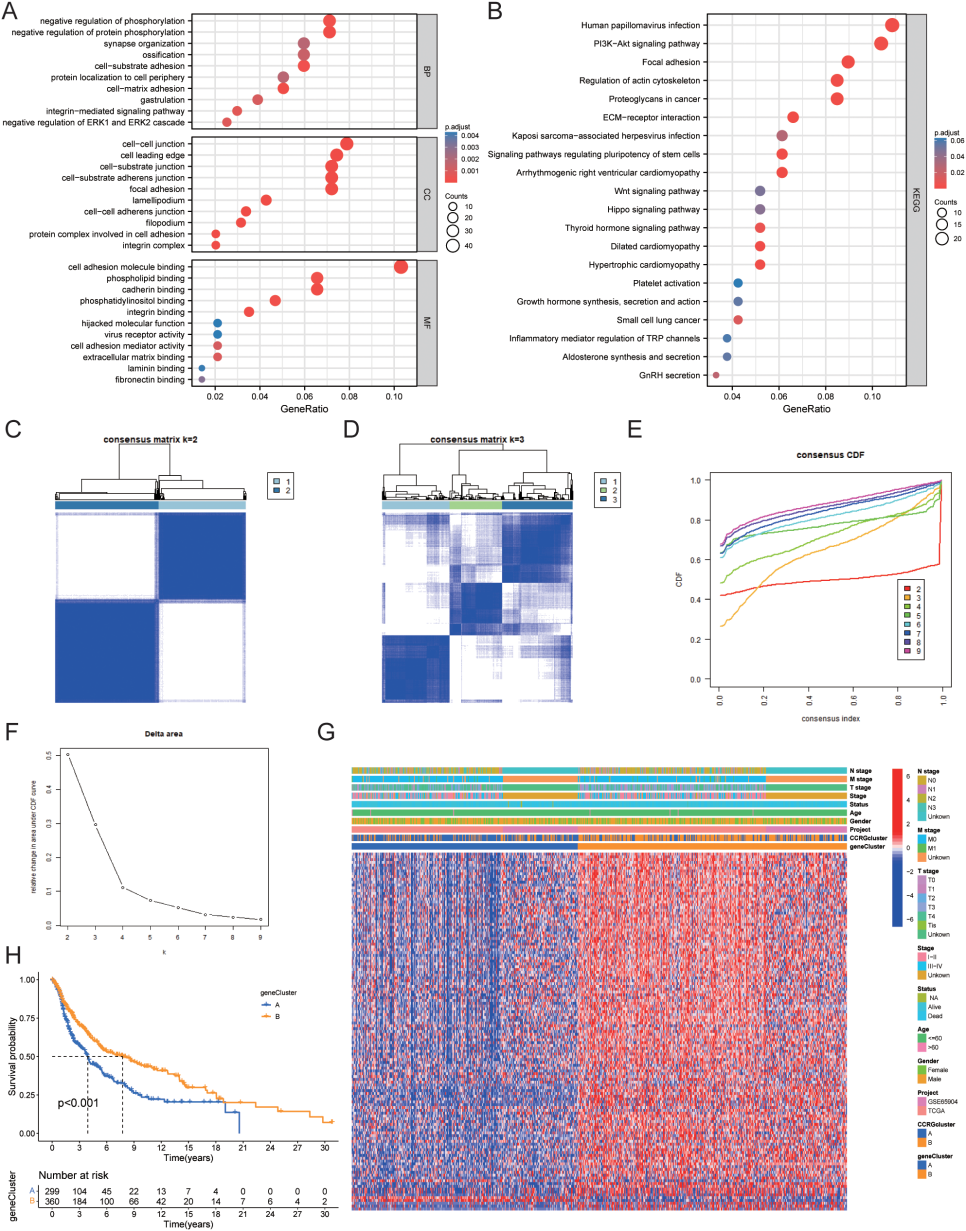
Identification of gene clusters based on OS-DEGs. **(A)** GO functional enrichment analysis of DEGs between circadian rhythm-related molecular subtypes. **(B)** KEGG functional enrichment analysis of DEGs between circadian rhythm-related molecular subtypes. **(C)** Consensus clustering heatmap defining two clusters (k = 2) and their associated area. **(D)** Consensus clustering heatmap defining three clusters (k = 3) and their associated area. **(E)** CDF plot showing the distribution of consensus clustering corresponding to each k. **(F)** Delta area plot visualizing the relative change in the area under the CDF curve at different k values. **(G)** Heatmap showing the relationship between gene expression levels and clinicopathological parameters across the two gene clusters. **(H)** Kaplan-Meier curve showing the prognostic differences between the two gene clusters. DEG, Differentially Expressed Gene; GO, Gene Ontology; KEGG, Kyoto Encyclopedia of Genes and Genomes; CDF, Cumulative Distribution Function.

Using the “limma” package in R, we found 457 circadian rhythm subtype-related DEGs and carried out functional enrichment analysis to investigate the possible biological roles of each CRG cluster. (**Figures 6A, B**, **Table 2**). Cell adhesion and communication-related biological processes, including cell-matrix adhesion, cell-substrate adhesion, cell-cell junction, integrin complex, cell adhesion molecule binding, and extracellular matrix binding, were shown to be enriched in DEGs, according to GO enrichment analysis. The ECM-receptor interaction, focal adhesion, proteoglycans in cancer, PI3K-Akt signaling pathway, and Hippo signaling pathway were among the pathways in which DEGs were enriched, according to KEGG enrichment analysis.

**Table 2:** Results of GO and KEGG enrichment analysis.

ONTOLOGY	ID	Description	p.adjust	Count
BP	GO:0007160	cell-matrix adhesion	6.21E-05	22
BP	GO:0001933	negative regulation of protein phosphorylation	6.21E-05	31
BP	GO:0042326	negative regulation of phosphorylation	0.000292	31
BP	GO:0031589	cell-substrate adhesion	0.000304	26
BP	GO:0007229	integrin-mediated signaling pathway	0.000693	13
BP	GO:0070373	negative regulation of ERK1 and ERK2 cascade	0.000781	11
BP	GO:0007369	gastrulation	0.001024	17
BP	GO:0001503	ossification	0.00138	26
BP	GO:0050808	synapse organization	0.00181	26
BP	GO:1990778	protein localization to cell periphery	0.00181	22
CC	GO:0031252	cell leading edge	8.26E-08	33
CC	GO:0005911	cell-cell junction	8.26E-08	35
CC	GO:0005925	focal adhesion	1.21E-07	32
CC	GO:0005924	cell-substrate adherens junction	1.21E-07	32
CC	GO:0030055	cell-substrate junction	1.23E-07	32
CC	GO:0008305	integrin complex	1.41E-06	9
CC	GO:0098636	protein complex involved in cell adhesion	2.95E-06	9
CC	GO:0005913	cell-cell adherens junction	3.52E-06	15
CC	GO:0030027	lamellipodium	4.17E-06	19
CC	GO:0030175	filopodium	4.17E-06	14
MF	GO:0050839	cell adhesion molecule binding	7.05E-11	44
MF	GO:0045296	cadherin binding	3.17E-06	28
MF	GO:0005178	integrin binding	0.000157	15
MF	GO:0005543	phospholipid binding	0.000297	28
MF	GO:0035091	phosphatidylinositol binding	0.000297	20
MF	GO:0050840	extracellular matrix binding	0.000917	9
MF	GO:0098631	cell adhesion mediator activity	0.001052	9
MF	GO:0001968	fibronectin binding	0.003071	6
MF	GO:0043236	laminin binding	0.004205	6
MF	GO:0001618	virus receptor activity	0.004282	9
MF	GO:0104005	hijacked molecular function	0.004282	9
KEGG	hsa04512	ECM-receptor interaction	9.8E-06	14
KEGG	hsa05412	Arrhythmogenic right ventricular cardiomyopathy	9.8E-06	13
KEGG	hsa04510	Focal adhesion	9.62E-05	19
KEGG	hsa05205	Proteoglycans in cancer	0.000399	18
KEGG	hsa04810	Regulation of actin cytoskeleton	0.000705	18
KEGG	hsa05165	Human papillomavirus infection	0.000705	23
KEGG	hsa05410	Hypertrophic cardiomyopathy	0.000731	11
KEGG	hsa05414	Dilated cardiomyopathy	0.001182	11
KEGG	hsa04550	Signaling pathways regulating pluripotency of stem cells	0.002465	13
KEGG	hsa04151	PI3K-Akt signaling pathway	0.003451	22
KEGG	hsa04919	Thyroid hormone signaling pathway	0.007015	11
KEGG	hsa05222	Small cell lung cancer	0.013198	9
KEGG	hsa04929	GnRH secretion	0.02517	7
KEGG	hsa05167	Kaposi sarcoma-associated herpesvirus infection	0.028658	13
KEGG	hsa04390	Hippo signaling pathway	0.044293	11
KEGG	hsa04310	Wnt signaling pathway	0.048091	11
KEGG	hsa04935	Growth hormone synthesis, secretion and action	0.054994	9
KEGG	hsa04925	Aldosterone synthesis and secretion	0.054994	8
KEGG	hsa04750	Inflammatory mediator regulation of TRP channels	0.058929	8
KEGG	hsa04611	Platelet activation	0.062936	9

We then performed univariate Cox regression analysis to determine the prognostic value of the 457 circadian rhythm subtype-related DEGs and identified 127 genes associated with overall survival (OS). We classified data into two gene clusters, A and B, using a consensus clustering technique based on the expression patterns of the 127 OS-related DEGs in order to further confirm this regulation mechanism. **Figure 6C** displays the consensus matrix calculated through consensus clustering, where k=2 successfully divides all samples into two distinct groups. The color intensity represents the similarity between samples, with darker colors indicating high similarity and lighter colors indicating greater differences. This result lays a foundation for subsequent analyses of potential biological functions. (**Figure 6D**) In the consensus matrix for k=3, the clustering of samples appears more complex, suggesting the potential presence of additional subgroups. Compared to k=2, the clustering quality shown here is lower, indicating that k=3 may not be suitable for effective sample classification. **Figure 6E** shows the consensus CDF curves for different k values. Observations reveal that the curve for k=2 has the highest proportion in the region above 0.8, indicating better clustering performance at this value. This further validates the effectiveness of k=2 as the optimal choice. **Figure 6F** indicates that as the k value increases, the largest drop in the Delta area occurs at k=2, supporting the use of k=2 for consensus clustering analysis. This clearly reflects k=2 as the optimal number of clusters. **Figure 6G** shows the relationship between OS-related DEG expression and clinicopathological features, with these genes being significantly more expressed in gene cluster B than in gene cluster A. Kaplan-Meier curves indicated that patients in gene cluster A had poorer OS, whereas gene cluster B exhibited longer OS (P<0.001) (**Figure 6H**).

### 3.6 Construction of a circadian rhythm-related prognostic model

**Figure 7:**
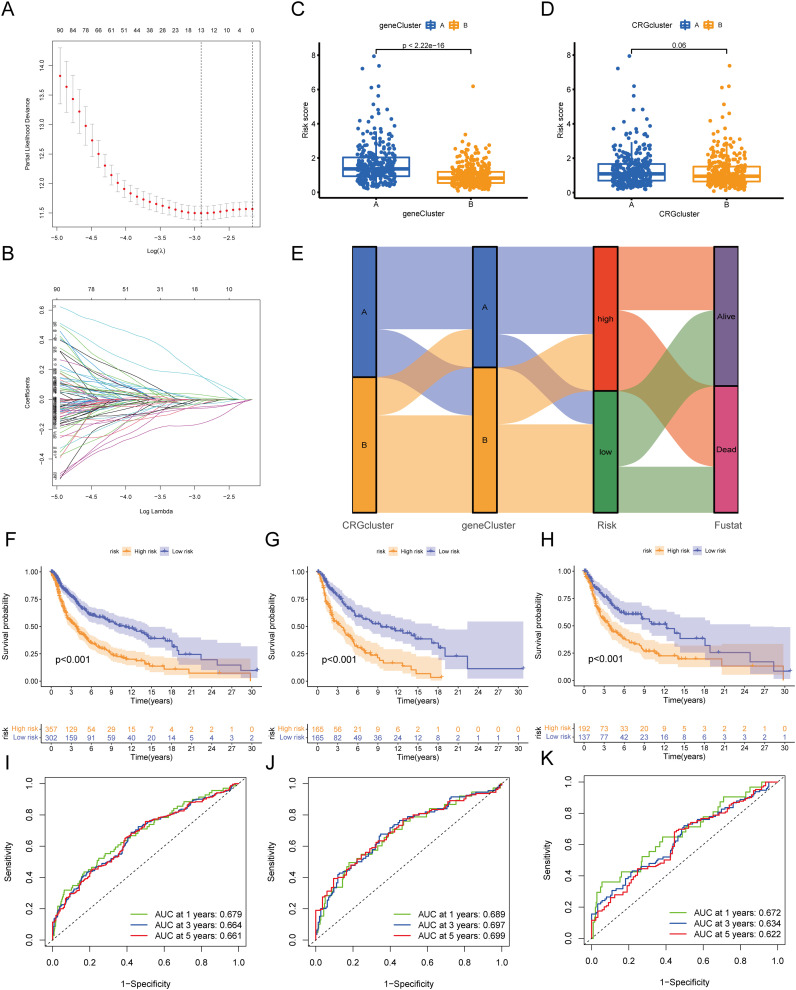
Construction of circadian rhythm prognostic model. **(A, B)** LASSO Cox regression analysis of OS-DEGs. **(C)** Differences in risk scores between gene cluster A and B. **(D)** Differences in risk scores between CRG cluster A and B. **(E)** Sankey diagram showing the relationships between molecular subtypes, gene clusters, risk scores, and survival status. **(F-H)** Kaplan-Meier curves showing prognostic differences between high and low-risk groups in the training set, validation set, and overall dataset. **(I-K)** ROC curves of the training set, validation set, and overall dataset. OS, Overall Survival; DEG, Differentially Expressed Gene; LASSO, Least Absolute Shrinkage and Selection Operator; ROC, Receiver Operating Characteristic Curve.

In this study, the training and validation sets used to construct the circadian rhythm-related prognostic model were randomly allocated in a 1:1 ratio after merging the TCGA and GEO datasets. This process ensured the representativeness of each dataset while providing a sufficient sample size for model construction and validation. LASSO and multivariate Cox regression analyses were conducted on the 127 OS-related DEGs to construct a prognostic model. Based on the smallest partial likelihood deviation in LASSO regression analysis, we retained 13 genes: *CMTM6*, *TNPO1*, *SLC5A3*, *CTBS*, *UTRN*, *HK2*, *SYNM*, *ZNF697*, *CSGALNACT1*, *GBP3*, *LIF*, *TPD52L1*, and *BCAN* (**Figures 7A, B**). Multivariate Cox regression analysis ultimately produced a prognostic model containing six genes, with the risk score calculated as follows: Riskscore=CMTM6*−0.286+TNPO1*0.523+CTBS*−0.361+UTRN*−0.209+HK2*0.308+LIF*−0.131 . We found a significant difference in risk scores between gene clusters A and B (P<2.2e-16), while no significant difference in risk scores was observed between CRG clusters A and B (**Figures 7C, D**). **Figure 7E** shows the relationship between molecular subtypes, gene clusters, risk scores, and survival status. Kaplan-Meier survival curves demonstrated that patients with low-risk scores had significantly better OS than those with high-risk scores in the training set, validation set, and overall dataset (**Figures 7F–H**). The ROC curves for the training set indicated that the model’s AUC for 1, 3, and 5 years was 0.679, 0.664, and 0.661, respectively. Additionally, ROC curves for the validation set and overall dataset also demonstrated good predictive performance of the model (**Figures 7I–K**).

### 3.7 Gene Set Enrichment Analysis

To further explore the functions of the risk score, we performed GSEA on the high-risk and low-risk groups. The results showed that the high-risk group was primarily enriched in pathways such as cristae formation, activation of the pre replicative complex, aminoacyl trna biosynthesis, gluconeogenesis, and DNA replication, as well as MYC_TARGETS_V2, hallmark myc targets v1, E2F targets, oxidative phosphorylation, and G2M checkpoint.

**Figure 8:**
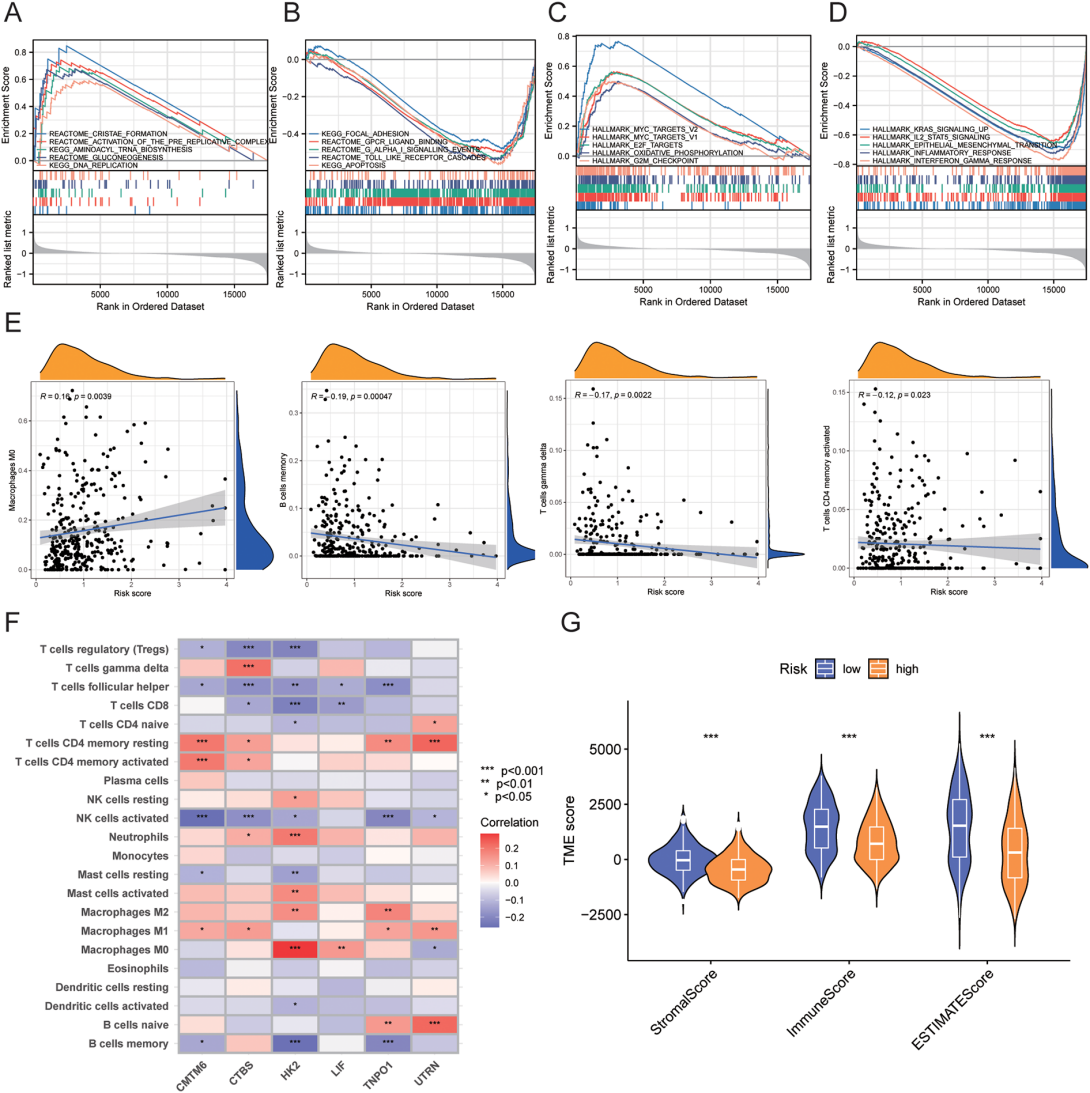
GSEA enrichment analysis and immune infiltration analysis between high-risk and low-risk groups. **(A-D)** GSEA enrichment analysis results for high-risk and low-risk groups. **(E)** Correlation between risk scores and immune cell infiltration levels. **(F)** Correlation between model genes and immune cell infiltration levels. **(G)** Correlation of TME scores between high-risk and low-risk groups. • indicates P<0.05, ** indicates P<0.01, *** indicates P<0.001. GSEA, Gene Set Enrichment Analysis; TME, Tumor Microenvironment.

In contrast, the low-risk group was mainly enriched in pathways such as focal adhesion, GPCR ligand binding, G alpha I signaling events, toll like receptor cascades, and apoptosis, as well as IL2 STAT5 signaling, epithelial mesenchymal transition, KRAS signaling up, inflammatory response, and interferon gamma response (**Figures 8A–D**, **Table 3**).

**Table 3:** Results of GSEA enrichment analysis.

Description	enrichmentScore	p.adjust
HALLMARK_MYC_TARGETS_V2	0.766095	0.026483
HALLMARK_MYC_TARGETS_V1	0.56333	0.080515
HALLMARK_E2F_TARGETS	0.561358	0.080515
HALLMARK_OXIDATIVE_PHOSPHORYLATION	0.499392	0.080515
HALLMARK_G2M_CHECKPOINT	0.497002	0.080515
HALLMARK_IL2_STAT5_SIGNALING	-0.64374	0.004145
HALLMARK_EPITHELIAL_MESENCHYMAL_TRANSITION	-0.65837	0.004145
HALLMARK_KRAS_SIGNALING_UP	-0.69475	0.004145
HALLMARK_INFLAMMATORY_RESPONSE	-0.72604	0.004145
HALLMARK_INTERFERON_GAMMA_RESPONSE	-0.77103	0.004145
KEGG_FOCAL_ADHESION	-0.48112	0.019213
REACTOME_GPCR_LIGAND_BINDING	-0.50436	0.019213
REACTOME_G_ALPHA_I_SIGNALLING_EVENTS	-0.53569	0.019213
REACTOME_TOLL_LIKE_RECEPTOR_CASCADES	-0.54188	0.019213
KEGG_APOPTOSIS	-0.56615	0.019213
REACTOME_CRISTAE_FORMATION	0.847113	0.037178
REACTOME_ACTIVATION_OF_THE_PRE_REPLICATIVE_COMPLEX	0.742989	0.046003
KEGG_AMINOACYL_TRNA_BIOSYNTHESIS	0.692399	0.04434
REACTOME_GLUCONEOGENESIS	0.677523	0.049903
KEGG_DNA_REPLICATION	0.589663	0.049903

### 3.8 Immune infiltration analysis between high-risk and low-risk groups

We assessed the relationship between risk scores and immune cell abundance using the CIBERSORTx algorithm. As shown in **Figure 8E**, risk scores were positively correlated with M0 macrophages and negatively correlated with memory B cells, gamma delta T cells, and activated CD4+ memory T cells. We also evaluated the relationship between immune cell abundance and the six genes in the prognostic model, finding that most immune cells, particularly HK2, were significantly correlated with these genes (P<0.05) (**Figure 8F**). We evaluated TME scores (stromal score, immune score, and ESTIMATE score) between high-risk and low-risk groups using the ESTIMATE package. We discovered that the high-risk group had significantly lower stromal score (P<0.001), immune score (P<0.001), and ESTIMATE score (P<0.001) than the low-risk group. (**Figure 8G**).

### 3.9 Prediction of immunotherapy response and drug sensitivity

**Figure 9:**
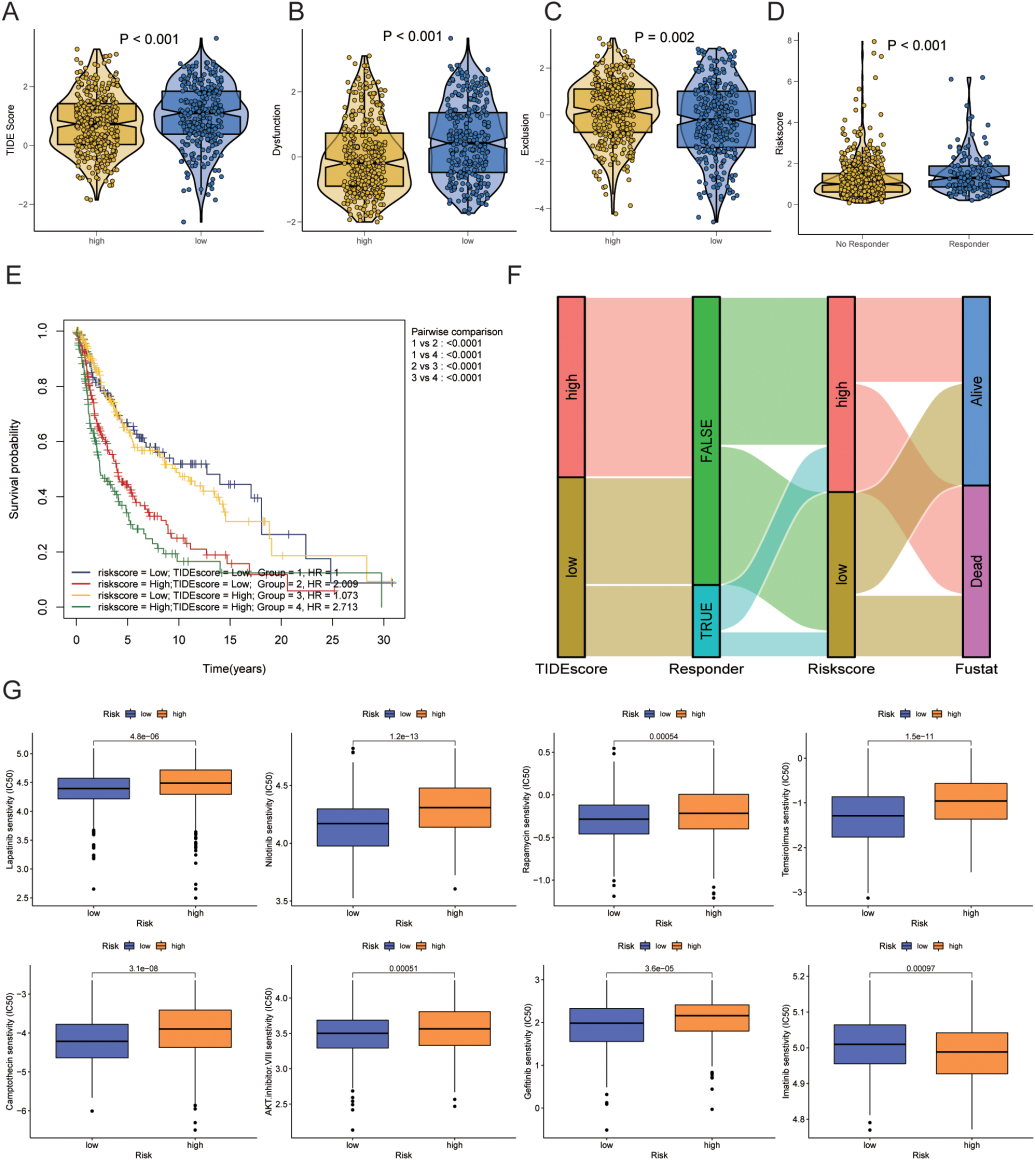
Prediction of immunotherapy response and drug sensitivity. **(A)** Differences in TIDE scores between high-risk and low-risk groups. **(B)** Differences in Dysfunction scores between high-risk and low-risk groups. **(C)** Differences in Exclusion scores between high-risk and low-risk groups. **(D)** Differences in risk scores between immunotherapy responders and non-responders as predicted by TIDE. **(E)** Kaplan-Meier curve combining risk scores and TIDE scores. **(F)** Sankey diagram showing the relationships between TIDE scores, immunotherapy response, risk scores, and survival status. **(G)** Prediction of drug sensitivity between high-risk and low-risk groups. TIDE, Tumor Immune Dysfunction and Exclusion.

We used TIDE scores to evaluate the effectiveness of immunotherapy. We found that, compared to the low-risk group, patients in the high-risk group had lower TIDE scores (P<0.001), lower Dysfunction scores (P<0.001), and higher Exclusion scores (P=0.002) (**Figures 9A–C**). According to the TIDE algorithm, immunotherapy responders had higher risk scores (P<0.001) (**Figure 9D**). Kaplan-Meier curves indicated that patients with both high-risk scores and high TIDE scores had the worst prognosis, whereas those with low-risk scores and low TIDE scores had the best prognosis (P<0.001) (**Figure 9E**). **Figure 9F** shows the relationships between TIDE scores, immunotherapy response, risk scores, and survival status.

Next, we analyzed differences in chemotherapy drug sensitivity between the high-risk and low-risk groups. The high-risk group exhibited decreased sensitivity (increased IC50) to AKT inhibitor VIII (P=0.00051), Camptothecin (P=3.1e-08), Gefitinib (P<0.001), Lapatinib (P=3.6e-05), Nilotinib (P=1.2e-13), Rapamycin (P=0.00054), and Temsirolimus (P=1.5e-11). In contrast, the sensitivity to Imatinib (P=0.00097) was increased (decreased IC50) in the high-risk group (**Figure 9G**).

### 3.10 Construction of a nomogram scoring system

**Figure 10:**
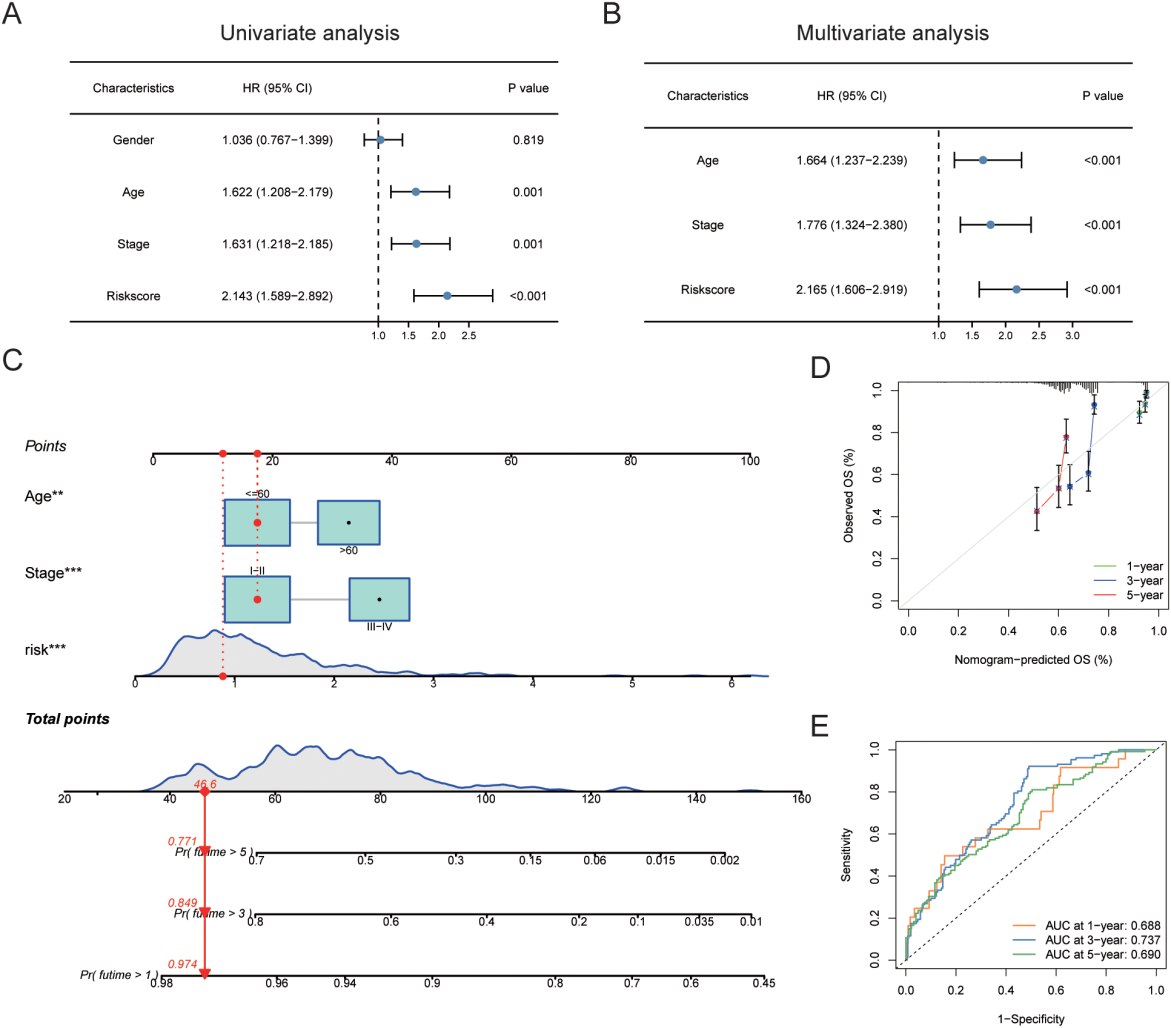
Construction of nomogram scoring system. **(A)** Results of univariate Cox analysis of risk scores and clinicopathological factors. **(B)** Results of multivariate Cox analysis of risk scores and clinicopathological factors. **(C)** Construction of a Nomogram scoring system to predict 1-year, 3-year, and 5-year survival. **(D)** Calibration curve of the Nomogram. **(E)** ROC curve of the Nomogram. ROC, Receiver Operating Characteristic Curve.

A univariate Cox regression analysis revealed a substantial correlation between risk scores and a patient’s bad prognosis. (HR [95% CI] = 2.143 [1.589-2.892], P<0.001). Risk ratings for SKCM patients were found to be an independent unfavorable prognostic factor by multivariate Cox regression analysis. (HR [95% CI] = 2.165 [1.606-2.919], P<0.001) (**Figures 10A, B**, **Table 4**). Considering that the circadian rhythm risk score is not convenient for clinical application in predicting OS in SKCM patients, we developed a nomogram incorporating risk scores and clinicopathological parameters to predict 1-year, 3-year, 5-year, and 10-year OS. The predictive factors included risk score, patient age, and stage (**Figure 10C**). Calibration curves demonstrated good predictive accuracy of the model (**Figure 10D**). Time-dependent ROC results showed that the AUC values of the nomogram in the training group for 1, 3, 5, and 10 years were 0.688, 0.737, and 0.690, respectively (**Figure 10E**).

**Table 4:** Results of univariate and multivariate Cox analysis.

Characteristics	Total(N)	Univariate analysis		Multivariate analysis	
		Hazard ratio (95% CI)	P value	Hazard ratio (95% CI)	P value
Gender	413				
Female	157	Reference			
Male	256	1.036 (0.767-1.399)	0.819		
Age	413				
<=60	218	Reference			
>60	195	1.622 (1.208-2.179)	0.001	1.664 (1.237-2.239)	<0.001
Stage	413				
I-II	224	Reference			
III-IV	189	1.631 (1.218-2.185)	0.001	1.776 (1.324-2.380)	<0.001
Riskscore	413				
low	182	Reference			
high	231	2.143 (1.589-2.892)	<0.001	2.165 (1.606-2.919)	<0.001

## 4 Discussion

SKCM is recognized for its aggressive nature, high metastatic potential, and considerable therapeutic challenges. According to the latest cancer statistics, an estimated 97,610 new cases and 7,990 deaths are expected in the United States in 2023, reflecting a continued upward trend in incidence (1). This type of melanoma has become one of the fastest-growing malignancies in Western countries, particularly due to increased UV exposure and genetic predispositions. Advancements in early detection and novel therapeutic strategies have significantly improved survival rates for patients diagnosed at an early stage of melanoma. However, the prognosis for patients with late-stage or metastatic melanoma remains dismal. While non-metastatic melanoma patients could expect a five-year survival rate of up to 99%, this rate plummets to approximately 25% once the disease has metastasized (38). The molecular pathogenesis of SKCM is intricate, involving multiple genetic alterations and signaling pathways. Mutations in key oncogenes such as BRAF, NRAS, and c-KIT activate pathways including MAPK/ERK and PI3K/AKT, which drive cellular proliferation, survival, and migration. Approximately 48% of melanoma cases harbor a BRAF mutation, with the BRAF V600E mutation being particularly prevalent (39). These mutations not only fuel tumor growth but also contribute to the immune evasion mechanisms by up-regulating PD-L1 expression and modulating the tumor microenvironment to suppress immune surveillance (40). In terms of treatment, the advent of immune checkpoint inhibitors, such as anti-PD-1 (nivolumab and pembrolizumab) and anti-CTLA-4 (ipilimumab) antibodies, has revolutionized the management of advanced melanoma. These therapies have significantly improved outcomes, with about 45% of patients experiencing durable responses. However, the treatment success is not uniform, with resistance developing in a substantial subset of patients (41, 42). Moreover, the management of immune-related adverse events, which could significantly impact the quality of life, remains a critical aspect of treatment strategies.

Circadian Rhythm Genes (CRGs) orchestrate a vast array of biological processes vital for organismal homeostasis, including the cell cycle, DNA repair mechanisms, and metabolic pathways, all critical in tumorigenesis and cancer progression. The core clock mechanism involves a transcription-translation feedback loop wherein CLOCK and BMAL1 heterodimers promote the expression of period (Per1/2/3) and cryptochrome (Cry1/2) genes. These proteins, in turn, regulate their expression by interacting with CLOCK and BMAL1, establishing a circadian rhythm that impacts various cellular functions (43, 44). In the context of oncology, disruptions in CRG expression notably influence tumor behavior, affecting proliferation, apoptosis, and metastasis across several cancer types. For instance, in breast cancer, altered expression of Per genes has been linked to increased cell proliferation and reduced apoptosis, thereby exacerbating cancer progression. Similarly, studies in colorectal cancer have identified mutations in various CRGs that correlate with tumor growth and poor patient prognosis due to disrupted circadian control over cell cycle and apoptosis (45, 46).

In SKCM, recent studies have elucidated that CRGs such as CLOCK and BMAL1 significantly influence various cellular functions including the tumor microenvironment, cell cycle, apoptosis regulation, DNA damage response, metabolic reprogramming, and immune evasion, thus affecting the response to therapy. These genes modulate the functionality of immune cells within the tumor microenvironment, potentially altering immunotherapy outcomes. For example, the circadian clock regulates the timing of UV exposure, which impacts the efficacy of DNA repair mechanisms and consequently influences melanoma risk and progression (47, 48). Furthermore, CRGs interact directly with key cell cycle and apoptosis regulators. Studies have demonstrated that CLOCK and BMAL1 affect cell proliferation by regulating the expression of Wee1, a kinase that inhibits cell cycle progression from G2 to M phase, suggesting a mechanism where disruptions in circadian rhythms could promote uncontrolled cell growth typical of melanoma (49). Additionally, the timing of DNA repair processes is controlled by circadian regulators, which modulate the expression and activity of various nucleotide excision repair enzymes. In SKCM, where UV-induced DNA damage is a critical risk factor, the efficiency of repair mechanisms during peak UV exposure times could significantly influence the risk of mutation accumulation and tumor initiation. Research has shown that the expression of XPA, a crucial DNA repair protein, is circadian-regulated, aligning DNA repair processes with periods of likely UV exposure (50). Moreover, melanoma cells exhibit unique metabolic profiles influenced by the circadian clock. CRGs like BMAL1 are involved in the regulation of oxidative phosphorylation and glycolysis pathways, which are often altered in cancer cells to meet their increased energy demands. Disruption of these pathways could create a metabolic environment that facilitates melanoma progression and resistance to therapy (51).

Our study rigorously explored the genetic and transcriptional landscapes of CRGs in SKCM using advanced bioinformatics tools. By analyzing comprehensive datasets from TCGA and GEO, we identified significant somatic mutations and CNVs in CRGs such as *RORB*, *PER3*, and *CLOCK*, which are associated with diverse clinical outcomes and immune infiltration patterns in SKCM. Upon comparing mRNA expression levels of CRGs in tumor and normal tissues, it was observed that the expression of the *TIMELESS* gene was elevated and associated with poor prognosis, confirming findings from previous studies. Zhao et al. have specifically noted that *TIMELESS* may regulate DNA replication and cell cycle-related genes, potentially influencing melanoma progression (52). In contrast, *RORA* expression was notably reduced in tumor tissues. Consistent with findings from the Benna study, this reduction and the presence of rs339972 C and rs10519097 T alleles of *RORA* were linked to a decreased risk of developing melanoma (53). Our prognostic model suggests that overexpression of PER predicts a poor prognosis in melanoma. The implicated mechanism involves the recognition of m6A-modified PER1 by the protein YTHDF2, which may accelerate melanoma progression (54). More importantly, using LASSO regression analysis to identify the smallest partial likelihood deviation, followed by multivariate Cox regression analysis, we ultimately developed a prognostic model comprising six genes. These genes are believed to play critical roles in the progression of CMM. *CMTM6* has been identified as a key regulator of immune evasion in tumor cells. It promotes tumor cell escape from immune surveillance by modulating the expression of immune checkpoint molecules, particularly programmed death-ligand 1 (PD-L1). *CMTM6* stabilizes PD-L1 on the surface of tumor cells by preventing its lysosomal degradation, thereby enhancing the tumor’s ability to suppress T cell-mediated immune responses in the tumor microenvironment. This mechanism underscores the pivotal role of *CMTM6* in the regulation of immune checkpoint pathways, which are critical in tumor immune evasion and resistance to immunotherapy (55, 56). *CMTM6* also influences the interaction between tumor cells and immune cells within the tumor microenvironment. Studies suggest that *CMTM6* may modulate the activity of tumor-associated macrophages (TAMs), natural killer (NK) cells, and dendritic cells, thereby shaping the immune landscape of the tumor microenvironment. For instance, *CMTM6*-mediated stabilization of PD-L1 can inhibit the activation of cytotoxic NK cells and impair the antigen-presenting function of dendritic cells, further promoting an immunosuppressive microenvironment conducive to tumor growth and progression (57). The clinical significance of CMTM6 lies in its potential as a prognostic biomarker and therapeutic target. Elevated expression of CMTM6 has been associated with poor prognosis, including reduced survival rates and increased risk of tumor recurrence in several cancer types, such as lung cancer, melanoma, and colorectal cancer (58–60). Targeting CMTM6 has been proposed as a novel therapeutic strategy to enhance the efficacy of immune checkpoint blockade therapies by destabilizing PD-L1 and restoring T cell-mediated antitumor immunity. These findings highlight modulating *CMTM6* expression may offer new avenues for cancer immunotherapy, particularly in tumors where PD-L1 plays a critical role in immune evasion (59). *TNPO1* is a transport protein involved in nuclear-cytoplasmic trafficking which plays a critical role in regulating the cell cycle and is implicated in tumor cell proliferation and metastasis. Overexpression of *TNPO1* has been linked to increased tumor cell proliferation and migration, contributing to aggressive cancer phenotypes. For instance, elevated *TNPO1* expression has been observed in colorectal cancer, where it correlates with enhanced tumor growth and poor prognosis (61). *TNPO1* influences tumor progression through its involvement in critical oncogenic signaling pathways. It has been shown to regulate components of the PI3K/Akt and MAPK pathways, which are essential for tumor cell survival, proliferation, and migration. By modulating the nuclear transport of signaling proteins, *TNPO1* indirectly affects downstream signaling cascades that drive tumor growth and metastasis (61). *TNPO1* is a key mediator of nucleocytoplasmic transport, specifically facilitating the nuclear import of RNA and proteins critical for cellular homeostasis. In tumor cells, *TNPO1* plays a pivotal role in the transport of transcription factors, splicing regulators, and other nuclear-localized proteins that govern gene expression and cellular behavior. This function is particularly relevant in cancer, as dysregulated nuclear transport can lead to aberrant gene expression patterns that promote tumor growth, invasion, and metastasis (62). Additionally, *TNPO1*-mediated transport may contribute to the adaptation of tumor cells to their microenvironment, enhancing their ability to evade immune surveillance and thrive in metastatic sites (62, 63). Moreover, *TNPO1* has been linked to melanoma cell proliferation and metastasis through its regulation of nuclear import/export processes. Its dysregulation is associated with poor prognosis in cancers (64). Cathepsin B serine protease is involved in extracellular matrix remodeling and tumor invasion. Elevated expression of *CTBS* facilitates melanoma metastasis by promoting cell migration and invasiveness (65). A key glycolytic enzyme, hexokinase 2 (*HK2*) is associated with metabolic reprogramming in melanoma cells. Its upregulation promotes the Warburg effect, which supports tumor growth and survival (66). *SLC5A3* plays a central role in ion transport and energy metabolism. Its expression levels may directly impact the metabolic state of cells, thereby influencing tumor growth and metastatic potential (67). These investigations extend beyond previous studies by not only identifying these variations but also linking them to functional consequences within the tumor microenvironment. The methodological approach of this study stands out due to its use of consensus clustering to classify SKCM patients into distinct molecular subtypes, CRG cluster A and B, based on CRG expression profiles.

The GSVA analysis results revealed that CRG cluster B was significantly enriched with several pathways closely associated with cellular biological functions. For example, in Protein Synthesis and Metabolism: the “GOBP_POSITIVE_REGULATION_OF_RESPONSE_TO_ENDOPLASMIC_RETICULUM_STRESS” pathway is involved in regulating the cellular response to endoplasmic reticulum (ER) stress. This pathway may be linked to the survival and adaptability of melanoma cells under external stress conditions (68). What’s more, Transport and Signal Transduction: The “KEGG_MTOR_SIGNALING_PATHWAY” is closely associated with cell growth, proliferation, and survival. This pathway is a critical regulator of tumor cell metabolism and drug resistance, playing a pivotal role in melanoma progression (69). Correspondingly, recent research has corroborated that selective inhibition of these pathways(mTOR and ERBB) presents a promising therapeutic avenue for melanoma variants that exhibit resistance to PD-1 monoclonal antibodies, which are critical in the disease’s advancement (70, 71). Biosynthesis and Repair Pathways: Pathways such as “GOBP_GOLGI_ORGANIZATION” and “GOBP_MAGNESIUM_ION_TRANSPORT” suggest that alterations in endoplasmic reticulum and Golgi apparatus functions may support the biosynthetic and repair processes required for tumor growth in melanoma cells (72). Moreover, The GSEA analysis results revealed significant differences in biological processes and signaling pathways between the high-risk and low-risk groups. Pathways Enriched in the High-Risk Group: The high-risk group was primarily enriched in pathways such as “REACTOME_ACTIVATION_OF_THE_PRE_REPLICATIVE_COMPLEX” and “KEGG_DNA_REPLICATION.” These findings suggest that enhanced cell cycle regulation and DNA replication in melanoma cells may contribute to the rapid proliferation of tumor cells. These pathways provide potential therapeutic targets, particularly for the use of cell cycle inhibitors in cancer treatment (73–75). Pathways Enriched in the Low-Risk Group: The low-risk group exhibited enrichment in pathways such as “KEGG_FOCAL_ADHESION” and “REACTOME_GPCR_LIGAND_BINDING.” This indicates that patients in the low-risk group may have stronger cell-cell interactions and adhesion abilities. These findings imply that tumors in the low-risk group are more effectively integrated within the tumor microenvironment, potentially linked to local immune responses or tumor suppression (76, 77). Targeting these pathways could potentially disrupt the melanoma’s progression mechanisms, offering new hope for treatment-resistant forms of this malignancy. Furthermore, the immune infiltration analysis demonstrated that CRG cluster A had higher infiltration of immune cells such as activated CD8+ T cells and macrophages, which are known to be critical for anti-tumor immunity, compared to CRG cluster B. The differential immune infiltration between high-risk and low-risk groups provides insights into the prognostic value of CRGs in SKCM. High-risk patients, as identified by our prognostic model, exhibited lower levels of immune cell infiltration, which might contribute to their poorer prognosis. Conversely, low-risk patients showed higher immune scores, indicating a more robust immune surveillance mechanism. In our study, we observed significant differences in immune infiltration between the high-risk and low-risk groups, which may have important implications for the effectiveness of immunotherapy. In the high-risk group, lower levels of immune cell infiltration may reflect a microenvironment characterized by immune evasion mechanisms, such as the upregulation of immune checkpoint molecules or the recruitment of immunosuppressive cells, such as regulatory T cells (Tregs) and myeloid-derived suppressor cells (MDSCs). These mechanisms are known to diminish immune system activity, potentially leading to reduced responses to immunotherapy and weakened immune surveillance, allowing tumor cells to evade recognition by the immune system (78, 79). The immune microenvironment in the high-risk group suggests that these patients may respond poorly to conventional immune checkpoint inhibitors (e.g., PD-1/PD-L1 inhibitors). This is consistent with studies that have demonstrated that immunosuppressive microenvironments reduce the efficacy of immunotherapy. Furthermore, the increased presence of MDSCs may exacerbate immune suppression by secreting immunosuppressive cytokines, thereby further impairing anti-tumor immune responses and enhancing tumor immune evasion. In contrast, the low-risk group exhibits higher immune cell infiltration, which may indicate a more active and effective anti-tumor immune response and likely to support more robust anti-tumor responses, making these patients more sensitive to immunotherapy. Elevated levels of effector T cells and natural killer (NK) cells suggest stronger immune surveillance, which has been associated with improved responses to immune checkpoint blockade therapies (80). These findings underscore the potential for stratifying patients based on immune infiltration patterns to predict immunotherapy outcomes and optimize treatment strategies. Further analysis of these differences could enhance our understanding of the tumor immune microenvironment and guide the development of combination therapies that target immune evasion mechanisms in high-risk patients, improving their response to treatment. This finding aligns with the immunosuppressive nature of the TME in high-risk groups and suggests that CRG expression could be a key modulator of immune evasion in melanoma (81). CRGs such as *CLOCK* and *BMAL1* play pivotal roles in modulating the tumor immune microenvironment, influencing immune cell function and potentially contributing to immune evasion mechanisms. The *CLOCK* protein, a core component of the circadian clock, forms a heterodimer with *BMAL1* to regulate the expression of genes involved in various physiological processes, including immune responses. Disruption of *CLOCK* expression can lead to altered immune cell function, impacting the body’s ability to mount effective anti-tumor responses. Studies have shown that circadian disruptions can affect the recruitment and function of myeloid-derived suppressor cells (MDSCs), which facilitate immune evasion in tumors (82). On the other hand, *BMAL1* is integral to maintaining the circadian rhythm and has been implicated in the regulation of immune cell metabolism and function. In macrophages, *BMAL1* acts as a metabolic sensor, influencing inflammatory responses and phagocytic activity. Deficiency in *BMAL1* has been associated with reduced inflammatory responses and altered metabolic processes in microglial cells, suggesting its role in modulating immune cell activity within the tumor microenvironment (83). Disruptions in circadian clock genes like *CLOCK* and *BMAL1* can lead to immune dysregulation, creating an environment conducive to tumor progression. Abnormal circadian rhythms have been linked to upregulation of immune inhibitory molecules such as PD-L1 and CTLA-4, contributing to T cell exhaustion and immune evasion in cancer (84). According to Adegoke et al., patients whose tumors had a higher infiltration of immune cells, such as macrophages, experienced better response rates and longer progression-free survival compared to those with tumors characterized by moderate or scarce immune cell presence (81). Future research should focus on elucidating the precise mechanisms by which these CRGs influence immune cell infiltration and function in SKCM, potentially uncovering novel therapeutic targets to enhance anti-tumor immunity. The predictive accuracy of our risk model was further validated using ROC curve analysis, with the nomogram integrating clinical parameters and risk scores demonstrating substantial predictive power for 1-, 3-, and 5-year overall survival. Compared to other studies, such as those by Cabrita and Cirenajwis (14, 15), which predominantly focused on broader genomic profiling, our research specifically targets the circadian regulatory network. This unique focus allows us to provide novel insights into the temporal dynamics of gene expression in SKCM. Unlike general genomic studies that provide a static snapshot of genetic alterations, our approach delves into how these alterations are influenced by the body’s circadian rhythms, offering a dynamic perspective that is critical for understanding tumor behavior over time. In our drug sensitivity analysis, the sensitivity differences to AKT inhibitor VIII were particularly notable. Although AKT inhibitors have not yet become a standard part of melanoma treatment, their clinical potential should not be overlooked. The PI3K/AKT signaling pathway plays a crucial role in melanoma progression, making AKT a promising therapeutic target. Several AKT inhibitors have been developed and are undergoing clinical evaluation. For instance, ipatasertib, an oral AKT inhibitor, has shown potential in clinical trials targeting tumors with specific genetic alterations. Additionally, the combination of AKT inhibitors with other therapeutic agents is being explored to overcome resistance mechanisms and enhance treatment efficacy. For example, combining AKT inhibitors with BRAF inhibitors has demonstrated synergistic effects in preclinical melanoma models, suggesting a potential strategy for patients with BRAF-mutant melanoma (85). As a result, AKT inhibitors have shown potential therapeutic value in inhibiting tumor cell proliferation and promoting apoptosis (86). Currently, multiple AKT inhibitors are undergoing clinical trials to explore their specific applications in melanoma treatment. Among them, AKT inhibitor VIII has demonstrated efficacy in selectively targeting the AKT pathway and effectively suppressing melanoma cell proliferation, providing a promising avenue for personalized treatment strategies. Camptothecin, a topoisomerase I inhibitor, has shown anti-tumor activity in various cancers, including melanoma (87). However, due to its toxicity and solubility issues, derivatives such as irinotecan and topotecan have been developed and are currently used in clinical settings. These agents interfere with DNA replication in rapidly dividing cells, leading to cell death (88). While not standard treatments for melanoma, camptothecin derivatives are being investigated in clinical trials, either alone or in combination with other therapies, to assess their efficacy and safety profiles in melanoma patients (89). Our findings suggest that patients in the low-risk group may exhibit increased sensitivity to AKT inhibitors and camptothecin derivatives, potentially leading to better therapeutic outcomes. Conversely, high-risk patients may require alternative strategies or combination therapies to achieve optimal results. Incorporating these insights into clinical decision-making could enhance personalized treatment approaches for melanoma patients.

In this study, we performed differential expression analysis between tumor and normal groups using the limma package, a widely accepted and well-performing method that has been successfully applied in many high-throughput data analyses. Furthermore, our analysis was primarily focused on key biological hypotheses, and the differentially expressed genes identified have been partially validated in the literature (49, 53). Therefore, the primary objective of this study is to investigate the impact of circadian rhythms on tumorigenesis and progression, identify key genes associated with SKCM and their potential mechanisms, and develop a prognostic model. SNPs and CNVs, as genetic markers, can influence patients’ clinical manifestations through various mechanisms (90). These variations can lead to changes in gene function, thereby affecting disease progression and treatment responses at multiple levels. In SKCM patients, CNVs may result in the loss of key tumor suppressor genes or the activation of oncogenes, thus promoting tumor development. For example, the loss of tumor suppressor genes can disrupt cell cycle checkpoints, leading to faster cell proliferation (91, 92). SNPs may affect the function of key genes in signaling pathways, altering cell growth regulation and impacting tumor proliferation and progression (93). Certain SNPs and CNVs can modify the structure and function of drug targets or drug-metabolizing enzymes, directly influencing patients’ responses to specific drugs (94). Genetic variations can also affect immune cell infiltration, angiogenesis, and other factors, thereby altering the tumor microenvironment and influencing patient survival rates (95). Furthermore, our study lays the groundwork for more personalized approaches in cancer treatment, where circadian biomarkers could predict patient-specific optimal treatment times, enhancing survival rates and quality of life for melanoma patients. Although our study provides robust insights, it is not without limitations. The reliance on retrospective data and potential biases in the TCGA and GEO datasets could affect the generalizability of the findings. Moreover, functional validation of CRG-related mechanisms in experimental models is necessary to confirm the causative roles suggested by our analyses. Although the prognostic model we constructed demonstrates good predictive performance, the lack of external dataset validation may limit the generalizability and applicability of the results. Future research will consider incorporating independent datasets to enhance the comprehensiveness of the findings and we plan to collect clinical samples and employ experimental methods such as qRT-PCR or IHC to further validate the conclusions of this study. Last but not least, immune infiltration analysis was included as a complementary aspect to broaden the research perspective and provide insights into the role of circadian rhythm-related genes in skin cutaneous melanoma. It is critical to incorporate additional experimental validations in future studies to enhance the credibility and comprehensiveness of our research.

Our study’s integration of circadian genomics with immune profiling offers a groundbreaking perspective, suggesting that the timing of therapeutic interventions could be crucially optimized based on circadian influences. This innovative approach not only proposes the possibility of enhancing treatment efficacy but also suggests reducing side effects by aligning treatment schedules with the body’s biological clock. Such synchronization could exploit the natural peak activity phases of cancer cell vulnerability and immune system responsiveness, potentially transforming the strategic planning of melanoma therapy. The implications of our findings extend beyond the immediate benefits of therapy timing. By understanding the circadian modulation of gene expression, our research significantly contributes to the broader understanding of melanoma pathogenesis. This knowledge is pivotal for developing future interventions that are finely tuned to the biological rhythms of the body, thereby improving prognostic assessments and therapeutic outcomes.

## 5 Conclusion

This study systematically explored the role of CRGs in SKCM. Our analysis identified significant gene alterations and differential expression patterns of CRGs between tumor and normal tissues, revealing their association with poor prognosis and immune modulation. Two molecular subtypes were established based on CRG expression, showing distinct clinical features and immune infiltration patterns. A prognostic model comprising six key CRGs (e.g., CMTM6, TNPO1, and HK2) demonstrated reliable accuracy in predicting overall survival. High-risk scores were linked to elevated immune checkpoint expression, decreased immune cell infiltration, and lower sensitivity to chemotherapies, underscoring CRGs’ impact on immune evasion and therapy resistance.

## Data Availability

The original contributions presented in the study are included in the article/**Supplementary Material**. Further inquiries can be directed to the corresponding authors.

## Supplementary material

Supplementary Figure 1 PCA Plots Before and After Data Merging. **(A)** PCA plot before merging TCGA-SKCM and GSE65904 datasets. **(B)** PCA plot after merging TCGA-SKCM and GSE65904 datasets. PCA: Principal Component Analysis; TCGA: The Cancer Genome Atlas; SKCM: Skin Cutaneous Melanoma.

## References

[1] Siegel RL Miller KD Wagle NS Jemal A. Cancer statistics, 2023. CA Cancer J Clin. (2023) 73:17–48. doi: 10.3322/caac.21763 36633525

[2] Arnold M Singh D Laversanne M Vignat J Vaccarella S Meheus F. Global burden of cutaneous melanoma in 2020 and projections to 2040. JAMA Dermatol. (2022) 158:495–503. doi: 10.1001/jamadermatol.2022.0160 35353115 PMC8968696

[3] Long GV Swetter SM Menzies AM Gershenwald JE Scolyer RA. Cutaneous melanoma. Lancet. (2023) 402:485–502. doi: 10.1016/S0140-6736(23)00821-8 37499671

[4] Larkin J Minor D D’Angelo S Neyns B Smylie M Miller WH Jr. Overall survival in patients with advanced melanoma who received nivolumab versus investigator’s choice chemotherapy in checkMate 037: A randomized, controlled, open-label phase III trial. J Clin Oncol. (2018) 36:383–90. doi: 10.1200/JCO.2016.71.8023 PMC6804912 28671856

[5] Jenkins RW Fisher DE. Treatment of advanced melanoma in 2020 and beyond. J Invest Dermatol. (2021) 141:23–31. doi: 10.1016/j.jid.2020.03.943 32268150 PMC7541692

[6] Ruben MD Wu G Smith DF Schmidt RE Francey LJ Lee YY. A database of tissue-specific rhythmically expressed human genes has potential applications in circadian medicine. Sci Transl Med. (2018) 10:eaat8806. doi: 10.1126/scitranslmed.aat8806 30209245 PMC8961342

[7] Sulli G Rommel A Wang X Kolar MJ Puca F Saghatelian A. Pharmacological activation of REV-ERBs is lethal in cancer and oncogene-induced senescence. Nature. (2018) 553:351–5. doi: 10.1038/nature25170 PMC5924733 29320480

[8] Papagiannakopoulos T Bauer MR Davidson SM Heimann M Subbaraj L Bhutkar A. Circadian rhythm disruption promotes lung tumorigenesis. Cell Metab. (2016) 24:324–31. doi: 10.1016/j.cmet.2016.07.001 PMC5367626 27476975

[9] Jerigova V Zeman M Okuliarova M. Circadian disruption and consequences on innate immunity and inflammatory response. Int J Mol Sci. (2022) 23:13722. doi: 10.3390/ijms232213722 36430199 PMC9690954

[10] He Y Chen Y Dai X Huang S. Dysregulation of circadian clock genes associated with tumor immunity and prognosis in patients with colon cancer. Comput Math Methods Med. (2022) 2022:4957996. doi: 10.1155/2022/4957996 35880088 PMC9308515

[11] Markova-Car EP Jurišić D Ilić N Kraljević Pavelić S. Running for time: circadian rhythms and melanoma. Tumour Biol. (2014) 35:8359–68. doi: 10.1007/s13277-014-1904-2 24729125

[12] Hutter C Zenklusen JC. The cancer genome atlas: creating lasting value beyond its data. Cell. (2018) 173:283–5. doi: 10.1016/j.cell.2018.03.042 29625045

[13] GTEx Consortium. Human genomics. The Genotype-Tissue Expression (GTEx) pilot analysis: multitissue gene regulation in humans. Science. (2015) 348:648–60. doi: 10.1126/science.1262110 PMC4547484 25954001

[14] Cabrita R Lauss M Sanna A Donia M Skaarup Larsen M Mitra S. Tertiary lymphoid structures improve immunotherapy and survival in melanoma. Nature. (2020) 577:561–5. doi: 10.1038/s41586-019-1914-8 31942071

[15] Cirenajwis H Ekedahl H Lauss M Harbst K Carneiro A Enoksson J. Molecular stratification of metastatic melanoma using gene expression profiling: Prediction of survival outcome and benefit from molecular targeted therapy. Oncotarget. (2015) 6:12297–309. doi: 10.18632/oncotarget.3655 PMC4494939 25909218

[16] Ritchie ME Phipson B Wu D Hu Y Law CW Shi W. limma powers differential expression analyses for RNA-sequencing and microarray studies. Nucleic Acids Res. (2015) 43:e47. doi: 10.1093/nar/gkv007 25605792 PMC4402510

[17] Mayakonda A Lin DC Assenov Y Plass C Koeffler HP. Maftools: efficient and comprehensive analysis of somatic variants in cancer. Genome Res. (2018) 28:1747–56. doi: 10.1101/gr.239244.118 PMC6211645 30341162

[18] Mermel CH Schumacher SE Hill B Meyerson ML Beroukhim R Getz G. GISTIC2.0 facilitates sensitive and confident localization of the targets of focal somatic copy-number alteration in human cancers. Genome Biol. (2011) 12:R41. doi: 10.1186/gb-2011-12-4-r41 21527027 PMC3218867

[19] Feng D Xiong Q Zhang F Shi X Xu H Wei W. Identification of a novel nomogram to predict progression based on the circadian clock and insights into the tumor immune microenvironment in prostate cancer. Front Immunol. (2022) 13:777724. doi: 10.3389/fimmu.2022.777724 35154101 PMC8829569

[20] Takahashi JS Hong HK Ko CH McDearmon EL. The genetics of mammalian circadian order and disorder: implications for physiology and disease. Nat Rev Genet. (2008) 9:764–75. doi: 10.1038/nrg2430 PMC3758473 18802415

[21] Wilkerson MD Hayes DN. ConsensusClusterPlus: a class discovery tool with confidence assessments and item tracking. Bioinformatics. (2010) 26:1572–3. doi: 10.1093/bioinformatics/btq170 PMC2881355 20427518

[22] Liberzon A Birger C Thorvaldsdóttir H Ghandi M Mesirov JP Tamayo P. The Molecular Signatures Database (MSigDB) hallmark gene set collection. Cell Syst. (2015) 1:417–25. doi: 10.1016/j.cels.2015.12.004 PMC4707969 26771021

[23] Hänzelmann S Castelo R Guinney J. GSVA: gene set variation analysis for microarray and RNA-seq data. BMC Bioinf. (2013) 14:7. doi: 10.1186/1471-2105-14-7 PMC3618321 23323831

[24] Rooney MS Shukla SA Wu CJ Getz G Hacohen N. Molecular and genetic properties of tumors associated with local immune cytolytic activity. Cell. (2015) 160:48–61. doi: 10.1016/j.cell.2014.12.033 25594174 PMC4856474

[25] Bao M Wu A. Exploring anesthetic-induced gene expression changes and immune cell dynamics in atrial tissue post-coronary artery bypass graft surgery. Open Med (Wars). (2024) 19:20241014. doi: 10.1515/med-2024-1014 39156756 PMC11330158

[26] Gene Ontology Consortium. Gene Ontology Consortium: going forward. Nucleic Acids Res. (2015) 43:D1049–56. doi: 10.1093/nar/gku1179 PMC4383973 25428369

[27] Kanehisa M Goto S. KEGG: kyoto encyclopedia of genes and genomes. Nucleic Acids Res. (2000) 28:27–30. doi: 10.1093/nar/28.1.27 10592173 PMC102409

[28] Yu G Wang LG Han Y He QY. clusterProfiler: an R package for comparing biological themes among gene clusters. OMICS. (2012) 16:284–7. doi: 10.1089/omi.2011.0118 PMC3339379 22455463

[29] Simon N Friedman J Hastie T Tibshirani R. Regularization paths for cox’s proportional hazards model via coordinate descent. J Stat Softw. (2011) 39:1–13. doi: 10.18637/jss.v039.i05 PMC4824408 27065756

[30] Bao JH Li JB Lin HS Zhang WJ Guo BY Li JJ. Deciphering a novel necroptosis-related miRNA signature for predicting the prognosis of clear cell renal carcinoma. Anal Cell Pathol (Amst). (2022) 2022:2721005. doi: 10.1155/2022/2721005 35509814 PMC9061065

[31] Blanche P Dartigues JF Jacqmin-Gadda H. Estimating and comparing time-dependent areas under receiver operating characteristic curves for censored event times with competing risks. Stat Med. (2013) 32:5381–97. doi: 10.1002/sim.5958 24027076

[32] Subramanian A Tamayo P Mootha VK Mukherjee S Ebert BL Gillette MA. Gene set enrichment analysis: a knowledge-based approach for interpreting genome-wide expression profiles. Proc Natl Acad Sci U S A. (2005) 102:15545–50. doi: 10.1073/pnas.0506580102 PMC1239896 16199517

[33] Rusk N. Expanded CIBERSORTx. Nat Methods. (2019) 16:577. doi: 10.1038/s41592-019-0486-8 31249418

[34] Yoshihara K Shahmoradgoli M Martínez E Vegesna R Kim H Torres-Garcia W. Inferring tumour purity and stromal and immune cell admixture from expression data. Nat Commun. (2013) 4:2612. doi: 10.1038/ncomms3612 24113773 PMC3826632

[35] Fu J Li K Zhang W Wan C Zhang J Jiang P. Large-scale public data reuse to model immunotherapy response and resistance. Genome Med. (2020) 12:21. doi: 10.1186/s13073-020-0721-z 32102694 PMC7045518

[36] Yang W Soares J Greninger P Edelman EJ Lightfoot H Forbes S. Genomics of Drug Sensitivity in Cancer (GDSC): a resource for therapeutic biomarker discovery in cancer cells. Nucleic Acids Res. (2013) 41:D955–61. doi: 10.1093/nar/gks1111 PMC3531057 23180760

[37] Geeleher P Cox N Huang RS. pRRophetic: an R package for prediction of clinical chemotherapeutic response from tumor gene expression levels. PLoS One. (2014) 9:e107468. doi: 10.1371/journal.pone.0107468 25229481 PMC4167990

[38] SChadendorf D van Akkooi A Berking C Griewank KG Gutzmer R Hauschild A. Melanoma. Lancet. (2018) 392:971–84. doi: 10.1016/S0140-6736(18)31559-9 30238891

[39] Long GV Menzies AM Nagrial AM Haydu LE Hamilton AL Mann GJ. Prognostic and clinicopathologic associations of oncogenic BRAF in metastatic melanoma. J Clin Oncol. (2011) 29:1239–46. doi: 10.1200/JCO.2010.32.4327 21343559

[40] Hugo W Zaretsky JM Sun L Song C Moreno BH Hu-Lieskovan S. Genomic and transcriptomic features of response to anti-PD-1 therapy in metastatic melanoma. Cell. (2017) 168:542. doi: 10.1016/j.cell.2017.01.010 28129544

[41] Larkin J Chiarion-Sileni V Gonzalez R Grob JJ Cowey CL Lao CD. Combined nivolumab and ipilimumab or monotherapy in untreated melanoma. N Engl J Med. (2015) 373:23–34. doi: 10.1056/NEJMoa1504030 26027431 PMC5698905

[42] Weber JS D’Angelo SP Minor D Hodi FS Gutzmer R Neyns B. Nivolumab versus chemotherapy in patients with advanced melanoma who progressed after anti-CTLA-4 treatment (CheckMate 037): a randomised, controlled, open-label, phase 3 trial. Lancet Oncol. (2015) 16:375–84. doi: 10.1016/S1470-2045(15)70076-8 25795410

[43] Gaddameedhi S Selby CP Kaufmann WK Smart RC Sancar A. Control of skin cancer by the circadian rhythm. Proc Natl Acad Sci U S A. (2011) 108:18790–5. doi: 10.1073/pnas.1115249108 PMC3219110 22025708

[44] Sancar A Lindsey-Boltz LA Gaddameedhi S Selby CP Ye R Chiou YY. Circadian clock, cancer, and chemotherapy. Biochemistry. (2015) 54:110–23. doi: 10.1021/bi5007354 PMC4303322 25302769

[45] Fu L Pelicano H Liu J Huang P Lee C. The circadian gene Period2 plays an important role in tumor suppression and DNA damage response in *vivo* . Cell. (2002) 111:41–50. doi: 10.1016/s0092-8674(02)00961-3 12372299

[46] Mormont MC Waterhouse J Bleuzen P Giacchetti S Jami A Bogdan A. Marked 24-h rest/activity rhythms are associated with better quality of life, better response, and longer survival in patients with metastatic colorectal cancer and good performance status. Clin Cancer Res. (2000) 6:3038–45. 10955782

[47] Ruan W Yuan X Eltzschig HK. Circadian rhythm as a therapeutic target. Nat Rev Drug Discovery. (2021) 20:287–307. doi: 10.1038/s41573-020-00109-w 33589815 PMC8525418

[48] Janich P Toufighi K Solanas G Luis NM Minkwitz S Serrano L. Human epidermal stem cell function is regulated by circadian oscillations. Cell Stem Cell. (2013) 13:745–53. doi: 10.1016/j.stem.2013.09.004 24120744

[49] Wang Y Guo H He F. Circadian disruption: from mouse models to molecular mechanisms and cancer therapeutic targets. Cancer Metastasis Rev. (2023) 42:297–322. doi: 10.1007/s10555-022-10072-0 36513953

[50] Dakup P Gaddameedhi S. Impact of the circadian clock on UV-induced DNA damage response and photocarcinogenesis. Photochem Photobiol. (2017) 93:296–303. doi: 10.1111/php.12662 27861965 PMC5315601

[51] Padmanabhan K Billaud M. Desynchronization of circadian clocks in cancer: A metabolic and epigenetic connection. Front Endocrinol (Lausanne). (2017) 8:136. doi: 10.3389/fendo.2017.00136 28674522 PMC5474466

[52] Zhao S Wen S Liu H Zhou Z Liu Y Zhong J. High expression of TIMELESS predicts poor prognosis: A potential therapeutic target for skin cutaneous melanoma. Front Surg. (2022) 9:917776. doi: 10.3389/fsurg.2022.917776 36034394 PMC9406824

[53] Benna C Rajendran S Spiro G Menin C Dall’Olmo L Rossi CR. Gender-specific associations between polymorphisms of the circadian gene RORA and cutaneous melanoma susceptibility. J Transl Med. (2021) 19:57. doi: 10.1186/s12967-021-02725-5 33549124 PMC7866430

[54] Yu J Chai P Xie M Ge S Ruan J Fan X. Histone lactylation drives oncogenesis by facilitating m(6)A reader protein YTHDF2 expression in ocular melanoma. Genome Biol. (2021) 22:85. doi: 10.1186/s13059-021-02308-z 33726814 PMC7962360

[55] Burr ML Sparbier CE Chan YC Williamson JC Woods K Beavis PA. CMTM6 maintains the expression of PD-L1 and regulates anti-tumour immunity. Nature. (2017) 549:101–5. doi: 10.1038/nature23643 PMC5706633 28813417

[56] Mezzadra R Sun C Jae LT Gomez-Eerland R de Vries E Wu W. Identification of CMTM6 and CMTM4 as PD-L1 protein regulators. Nature. (2017) 549:106–10. doi: 10.1038/nature23669 PMC6333292 28813410

[57] Zugazagoitia J Liu Y Toki M McGuire J Ahmed FS Henick BS. Quantitative assessment of CMTM6 in the tumor microenvironment and association with response to PD-1 pathway blockade in advanced-stage non-small cell lung cancer. J Thorac Oncol. (2019) 14:2084–96. doi: 10.1016/j.jtho.2019.09.014 PMC6951804 31605795

[58] Dai F Duan YL Feng Q Song SL Yang JL Lv T. CMTM6: A critical prognostic indicator in non-small cell lung cancer. J Cancer. (2024) 15:2373–9. doi: 10.7150/jca.93733 PMC10937279 38495487

[59] Yaseen MM Abuharfeil NM Darmani H. CMTM6 as a master regulator of PD-L1. Cancer Immunol Immunother. (2022) 71:2325–40. doi: 10.1007/s00262-022-03171-y PMC10991181 35294592

[60] Peng QH Wang CH Chen HM Zhang RX Pan ZZ Lu ZH. CMTM6 and PD-L1 coexpression is associated with an active immune microenvironment and a favorable prognosis in colorectal cancer. J Immunother Cancer. (2021) 9:e001638. doi: 10.1136/jitc-2020-001638 33579737 PMC7883863

[61] Yue A Chen M Dai S Zhang Y Wei W Fan L. Tastin promotes non-small-cell lung cancer progression through the ErbB4, PI3K/AKT, and ERK1/2 pathways. Exp Biol Med (Maywood). (2023) 248:519–31. doi: 10.1177/15353702221147566 PMC10281536 36691332

[62] van der Watt PJ Okpara MO Wishart A Parker MI Soares NC Blackburn JM. Nuclear transport proteins are secreted by cancer cells and identified as potential novel cancer biomarkers. Int J Cancer. (2022) 150:347–61. doi: 10.1002/ijc.33832 34591985

[63] Yang B Chen J Li X Zhang X Hu L Jiang S. TNPO1-mediated nuclear import of ARID1B promotes tumor growth in ARID1A-deficient gynecologic cancer. Cancer Lett. (2021) 515:14–27. doi: 10.1016/j.canlet.2021.05.016 34044070

[64] Xi Y Shen Y Wu D Zhang J Lin C Wang L. CircBCAR3 accelerates esophageal cancer tumorigenesis and metastasis via sponging miR-27a-3p. Mol Cancer. (2022) 21:145. doi: 10.1186/s12943-022-01615-8 35840974 PMC9284725

[65] Girotti MR Fernández M López JA Camafeita E Fernández EA Albar JP. SPARC promotes cathepsin B-mediated melanoma invasiveness through a collagen I/α2β1 integrin axis. J Invest Dermatol. (2011) 131:2438–47. doi: 10.1038/jid.2011.239 21850018

[66] Patra KC Hay N. Hexokinase 2 as oncotarget. Oncotarget. (2013) 4:1862–3. doi: 10.18632/oncotarget.1563 PMC3875752 24196563

[67] Wei Y Huang YH Skopelitis DS Iyer SV Costa A Yang Z. SLC5A3-dependent myo-inositol auxotrophy in acute myeloid leukemia. Cancer Discovery. (2022) 12:450–67. doi: 10.1158/2159-8290.CD-20-1849 PMC8831445 34531253

[68] Verfaillie T Salazar M Velasco G Agostinis P. Linking ER stress to autophagy: potential implications for cancer therapy. Int J Cell Biol. (2010) 2010:930509. doi: 10.1155/2010/930509 20145727 PMC2817393

[69] Laplante M Sabatini DM. mTOR signaling in growth control and disease. Cell. (2012) 149:274–93. doi: 10.1016/j.cell.2012.03.017 PMC3331679 22500797

[70] DuBose E Bevill SM Mitchell DK Sciaky N Golitz BT Dixon S. Neratinib, a pan ERBB/HER inhibitor, restores sensitivity of PTEN-null, BRAFV600E melanoma to BRAF/MEK inhibition. Front Oncol. (2024) 14:1191217. doi: 10.3389/fonc.2024.1191217 38854737 PMC11159048

[71] Qi X Chen Y Liu S Liu L Yu Z Yin L. Sanguinarine inhibits melanoma invasion and migration by targeting the FAK/PI3K/AKT/mTOR signalling pathway. Pharm Biol. (2023) 61:696–709. doi: 10.1080/13880209.2023.2200787 37092313 PMC10128503

[72] De Matteis MA Luini A. Exiting the golgi complex. Nat Rev Mol Cell Biol. (2008) 9:273–84. doi: 10.1038/nrm2378 18354421

[73] Blow JJ Dutta A. Preventing re-replication of chromosomal DNA. Nat Rev Mol Cell Biol. (2005) 6:476–86. doi: 10.1038/nrm1663 PMC2688777 15928711

[74] Henrikus SS Gross MH Willhoft O Pühringer T Lewis JS McClure AW. Unwinding of a eukaryotic origin of replication visualized by cryo-EM. Nat Struct Mol Biol. (2024) 31:1265–76. doi: 10.1038/s41594-024-01280-z PMC11327109 38760633

[75] Zeman MK Cimprich KA. Causes and consequences of replication stress. Nat Cell Biol. (2014) 16:2–9. doi: 10.1038/ncb2897 24366029 PMC4354890

[76] Mitra SK Hanson DA Schlaepfer DD. Focal adhesion kinase: in command and control of cell motility. Nat Rev Mol Cell Biol. (2005) 6:56–68. doi: 10.1038/nrm1549 15688067

[77] Harrison C. Seven-transmembrane receptors: One way only. Nat Rev Drug Discovery. (2009) 8:932. doi: 10.1038/nrd3056 19967798

[78] Strande NT Brnich SE Roman TS Berg JS. Navigating the nuances of clinical sequence variant interpretation in Mendelian disease. Genet Med. (2018) 20:918–26. doi: 10.1038/s41436-018-0100-y PMC6679919 29988079

[79] Tang F Li J Qi L Liu D Bo Y Qin S. A pan-cancer single-cell panorama of human natural killer cells. Cell. (2023) 186:4235–51.e20. doi: 10.1016/j.cell.2023.07.034 37607536

[80] Tsur N Menashe I Haviv YS. Risk factors before dialysis predominate as mortality predictors in diabetic maintenance dialysis patients. Sci Rep. (2019) 9:10633. doi: 10.1038/s41598-019-46919-w 31337801 PMC6650444

[81] Adegoke NA Gide TN Mao Y Quek C Patrick E Carlino MS. Classification of the tumor immune microenvironment and associations with outcomes in patients with metastatic melanoma treated with immunotherapies. J Immunother Cancer. (2023) 11:e007144. doi: 10.1136/jitc-2023-007144 37865395 PMC10603328

[82] Mok H Ostendorf E Ganninger A Adler AJ Hazan G Haspel JA. Circadian immunity from bench to bedside: a practical guide. J Clin Invest. (2024) 134:e175706. doi: 10.1172/JCI175706 38299593 PMC10836804

[83] Timmons GA Carroll RG O’Siorain JR Cervantes-Silva MP Fagan LE Cox SL. The circadian clock protein BMAL1 acts as a metabolic sensor in macrophages to control the production of pro IL-1β. Front Immunol. (2021) 12:700431. doi: 10.3389/fimmu.2021.700431 34858390 PMC8630747

[84] Wu Y Tao B Zhang T Fan Y Mao R. Pan-cancer analysis reveals disrupted circadian clock associates with T cell exhaustion. Front Immunol. (2019) 10:2451. doi: 10.3389/fimmu.2019.02451 31708917 PMC6821711

[85] Lassen A Atefi M Robert L Wong DJ Cerniglia M Comin-Anduix B. Effects of AKT inhibitor therapy in response and resistance to BRAF inhibition in melanoma. Mol Cancer. (2014) 13:83. doi: 10.1186/1476-4598-13-83 24735930 PMC4021505

[86] Dudzisz-Śledź M Kondracka M Rudzińska M Ae Zając Firlej W Sulejczak D. Mesenchymal chondrosarcoma from diagnosis to clinical trials. Cancers (Basel). (2023) 15:4581. doi: 10.3390/cancers15184581 37760551 PMC10527018

[87] Lian C Cao S Zeng W Li Y Su J Li J. RJT-101, a novel camptothecin derivative, is highly effective in the treatment of melanoma through DNA damage by targeting topoisomerase 1. Biochem Pharmacol. (2020) 171:113716. doi: 10.1016/j.bcp.2019.113716 31751535

[88] Ng CE Bussey AM Raaphorst GP. Inhibition of potentially lethal and sublethal damage repair by camptothecin and etoposide in human melanoma cell lines. Int J Radiat Biol. (1994) 66:49–57. doi: 10.1080/09553009414550941 8027612

[89] Pantazis P Kozielski A Rodriguez R Petry E Wani M Wall M. Therapeutic efficacy of camptothecin derivatives against human Malignant melanoma xenografts. Melanoma Res. (1994) 4:5–10. doi: 10.1097/00008390-199402000-00002 8032218

[90] Akhtar MM Micolucci L Islam MS Olivieri F Procopio AD. Bioinformatic tools for microRNA dissection. Nucleic Acids Res. (2016) 44:24–44. doi: 10.1093/nar/gkv1221 26578605 PMC4705652

[91] Lukacova E Hanzlikova Z Podlesnyi P Sedlackova T Szemes T Grendar M. Novel liquid biopsy CNV biomarkers in Malignant melanoma. Sci Rep. (2024) 14:15786. doi: 10.1038/s41598-024-65928-y 38982214 PMC11233564

[92] Broseghini E Venturi F Veronesi G Scotti B Migliori M Marini D. Exploring the common mutational landscape in cutaneous melanoma and pancreatic cancer. Pigment Cell Melanoma Res. (2025) 38:e13210. doi: 10.1111/pcmr.13210 39609109 PMC11681848

[93] Grochola LF Zeron-Medina J Mériaux S Bond GL. Single-nucleotide polymorphisms in the p53 signaling pathway. Cold Spring Harb Perspect Biol. (2010) 2:a001032. doi: 10.1101/cshperspect.a001032 20452958 PMC2857176

[94] Lee NH. Pharmacogenetics of drug metabolizing enzymes and transporters: effects on pharmacokinetics and pharmacodynamics of anticancer agents. Anticancer Agents Med Chem. (2010) 10:583–92. doi: 10.2174/187152010794474019 PMC3770187 21194402

[95] Fang J Lu Y Zheng J Jiang X Shen H Shang X. Exploring the crosstalk between endothelial cells, immune cells, and immune checkpoints in the tumor microenvironment: new insights and therapeutic implications. Cell Death Dis. (2023) 14:586. doi: 10.1038/s41419-023-06119-x 37666809 PMC10477350

