# Supplementary figures and images for: Circadian rhythm related genes identified through tumorigenesis and immune infiltration-guided strategies as predictors of prognosis, immunotherapy response, and candidate drugs in skin cutaneous malignant melanoma
Source: Front Immunol. 2025 Mar 21;16:1513750. doi: 10.3389/fimmu.2025.1513750 (PMC11968383; doi:10.3389/fimmu.2025.1513750)

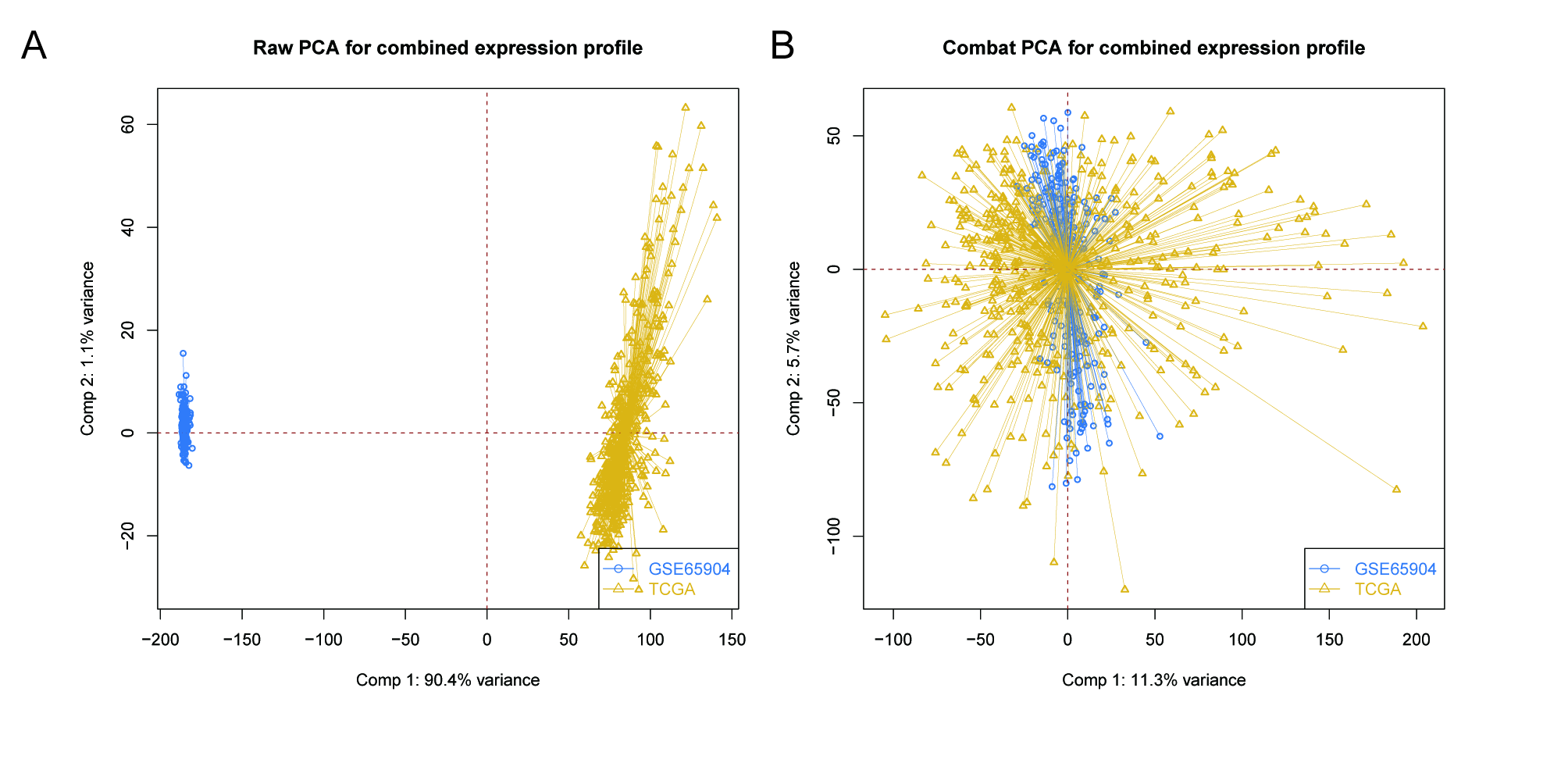

Supplement: Supplementary Figure 1 — PCA Plots Before and After Data Merging. (A) PCA plot before merging TCGA-SKCM and GSE65904 datasets. (B) PCA plot after merging TCGA-SKCM and GSE65904 datasets. PCA: Principal Component Analysis; TCGA: The Cancer Genome Atlas; SKCM: Skin Cutaneous Melanoma. [file Image1.tif]
